# Host Traits Impact the Outcome of Metagenomic Library Preparation From Dental Calculus Samples Across Diverse Mammals

**DOI:** 10.1111/1755-0998.70039

**Published:** 2025-09-01

**Authors:** Markella Moraitou, John L. Richards, Chanah Bolyos, Konstantina Saliari, Emmanuel Gilissen, Zena Timmons, Andrew C. Kitchener, Olivier S. G. Pauwels, Richard Sabin, Phaedra Kokkini, Roberto Portela Miguez, Katerina Guschanski

**Affiliations:** ^1^ Institute of Ecology and Evolution, School of Biological Sciences University of Edinburgh Edinburgh UK; ^2^ Natural History Museum Vienna Vienna Austria; ^3^ Royal Museum for Central Africa Tervuren Belgium; ^4^ Alzheimer and Other Tauopathies Research Group, ULB Center for Diabetes Research (UCDR) Université Libre de Bruxelles Brussels Belgium; ^5^ Department of Natural Sciences National Museums Scotland Edinburgh UK; ^6^ School of Geosciences University of Edinburgh Edinburgh UK; ^7^ Royal Belgian Institute of Natural Sciences Brussels Belgium; ^8^ Natural History Museum London London UK; ^9^ Department of Ecology and Genetics/Animal Ecology Uppsala University Uppsala Sweden

**Keywords:** ancient DNA, library preparation, museum collections, oral microbiome, shotgun sequencing

## Abstract

Dental calculus metagenomics has emerged as a valuable tool for studying the oral microbiomes of humans and a few select mammals. With increasing interest in wild animal microbiomes, it is important to understand how widely this material can be used across the mammalian tree of life, refine the related protocols and understand the expected outcomes and potential challenges of dental calculus sample processing. In this study, we significantly expand the breadth of studied host species, analysing laboratory and bioinformatics metadata of dental calculus samples from 32 ecologically and phylogenetically diverse mammals. Although we confirm the presence of an oral microbiome signature in the metagenomes of all studied mammals, the fraction recognised as oral varies between host species, possibly because of both biological differences and methodological biases. The overall success rate of dental calculus processing, from extractions to sequencing, was ~74%. Although input sample weight was positively associated with the number of produced library molecules, we identify a negative impact of enzymatic inhibition on the library preparation protocol. The inhibition was most prevalent in herbivores and frugivores and is likely diet‐derived. In contrast, hosts with an animalivore diet posed fewer challenges during laboratory processing and yielded more DNA relative to sample weight. Our results translate into recommendations for future studies of dental calculus metagenomics from a variety of host species, identifying required sample amounts, and emphasising the utility of dental calculus in exploring the oral microbiome in relation to broader ecological and evolutionary questions.

## 1 Introduction

Host‐associated microbiomes contribute to a multitude of fundamental biological functions of their hosts. The seemingly endless list includes processes as diverse as immune response (Ippolito et al. 2018, 202), reproduction (Comizzoli et al. 2021), nutrient metabolism (Rowland et al. 2018) and thermoregulation (Chevalier et al. 2015). As most studies focus on the human gut, the microbiomes of non‐human animals, especially of body sites other than the gut, remain largely understudied. However, it is unclear how far the knowledge from the human gut microbiome extends to the wider biological diversity of hosts and microbial communities. Animal microbiomes can have important implications for conservation and adaptation (Trevelline et al. 2019; Alberdi et al. 2016), act as reservoirs for zoonotic diseases (White and Razgour 2020) and respond to anthropogenic impacts, such as habitat degradation (Barelli et al. 2015; Amato et al. 2019) and contamination (Lapanje et al. 2010; Costa et al. 2016; Brealey et al. 2021).

Although less well studied than the gut microbiome, the oral microbiome has systemic effects on its host. It is involved in a diversity of systemic diseases (Nakano et al. 2009; Teles and Wang 2011; Olsen and Singhrao 2015; Flynn et al. 2016; Gao et al. 2016; Warinner 2016) and plays an important role in the production of nitric oxide (Duncan et al. 1995), a molecule that participates in blood flow regulation and inflammation (Ghimire et al. 2017). There is strong evidence that, through this process, the oral microbiome affects cardiovascular health (Blekkenhorst et al. 2018) and may even contribute to muscle oxygenation and, ultimately, exercise performance (Bryan et al. 2022). At the same time, the oral cavity is more exposed to the external environment than the gut microbiome and likely contains environmental microbes (Shaiber et al. 2020), which may offer additional insights into the host's ecology. Yet, collecting samples from wild animals remains challenging because of hurdles both logistic (fieldwork in remote locations, need for research permits, etc.) and ethical (potentially affecting the health and welfare of rare or endangered species). This is particularly true for microbiomes other than the gut. Whereas faecal samples can often be easily obtained without disturbance to the host individuals, sampling from other body sites often requires handling. Studying captive animals may lead to biased inferences, as captivity drastically alters and homogenises the microbiome, at least in the case of the gut (McKenzie et al. 2017; Clayton et al. 2016).

Dental calculus, the mineralised form of the dental plaque microbiome, offers a way to overcome many of the challenges presented above. Dental calculus forms on the surface of teeth through periodic mineralisations (Schroeder and Shanley 1969; Jepsen et al. 2011) of the microbial biofilm, entrapping and preserving the DNA of the oral microbiome, the host, and dietary components (Mann et al. 2018, 2020; Warinner et al. 2015). It is widespread among mammals and readily available from museum specimens (Brealey et al. 2020; Richards et al. 2025) and archaeological material (Warinner et al. 2014; Armitage 1975; Ciochon et al. 1990; Dobney and Brothwell 1987), which allows researchers to circumvent the logistic and ethical difficulties of handling wild animals. In addition, because of the exceptional preservation of genetic material (Mann et al. 2018; Velsko et al. 2019; Fellows Yates et al. 2021), this ‘microbial fossil’ can be sampled decades, centuries, or even millennia after the host's death, and analysed using metagenomics, providing snapshots of past microbiomes (Warinner et al. 2014). This has allowed for temporal studies of the oral microbiome, helping to understand changes in microbiome structure and function in the distant past (Quagliariello et al. 2022; Velsko et al. 2024; Gancz et al. 2023) or more recently, under increasing human impact on the environment (Brealey et al. 2021). Beyond its primary use for microbiome research, dental calculus can be used for host genomics. Because the eukaryotic content is low in this material (< 1%; Mann et al. 2018), shotgun metagenomics provides only a limited view of the host, but may still allow for molecular sexing, mitochondrial DNA assemblies, and low coverage host genomics (Brealey et al. 2020; Moraitou et al. 2022; Gower et al. 2019). Alternatively, researchers may opt for target enrichment techniques to increase host content (Ozga et al. 2016; Ziesemer et al. 2019).

Despite all these possible applications, dental calculus has so far been predominantly used to study the oral microbiome of humans (see Fotakis et al. 2020; Velsko et al. 2024; Gancz et al. 2023; Warinner 2016; Granehäll et al. 2021; Klapper et al. 2023, among others) and a small number of non‐human species (Moraitou et al. 2022; Brealey et al. 2021, 2020; Fellows Yates et al. 2021; Ozga and Ottoni 2021; Ottoni et al. 2019). Given its broad presence in mammals and the wide scope of questions in ecology and evolution that can be addressed with this material, we can expect its increased use in the future. Therefore, we need to better understand which factors determine the success of molecular analyses based on dental calculus. Although its sampling is usually less damaging to a specimen than sampling teeth or bone, it is still considered destructive, as the sampled material is used up during laboratory processing. Although dental calculus reportedly preserves DNA better than dentine (Mann et al. 2018), only 70%–80% of samples produce usable metagenomic data, with some evidence of sample enzymatic inhibition (personal observations). Therefore, understanding which sample characteristics influence success rate in the laboratory can help researchers plan their studies, minimising unnecessary sampling of rare museum specimens and better managing project resources.

This study aims to assess the efficiency and success rate of Illumina metagenomic library preparation from dental calculus, depending on the characteristics of the collected samples (such as dental calculus amount, DNA extract pigmentation, specimen age) and the host species (diet, taxonomic group). In the absence of any inhibition, we expect that sample weight will be positively correlated with DNA concentration in a standardised extraction and with the quantity of fragments that are successfully turned into next generation sequencing libraries. However, we hypothesise that additional factors, such as host diet, may impact the outcome of these processes. For instance, plants can contain secondary compounds, such as polyphenols, polysaccharides, tannins, etc., that inhibit enzymatic reactions (Schrader et al. 2012) and are often co‐extracted during sample processing, generating brown or yellow DNA extracts (Stevenson 1994). Therefore, we expect that dental calculus from herbivorous species with high content of such compounds in their diet may exhibit enzymatic inhibition, negatively affecting laboratory protocols. To test our hypotheses, we consider each step of the laboratory protocol (extractions, adapter ligations, and indexing) and assess the effect of host diet and taxonomy, extract pigmentation (suggestive of co‐eluted secondary compounds), and sample input amount on protocol success rate. Finally, we assess if different types of samples differ with regard to the proportion of the metagenome that resembles oral microbiomes and the host DNA content. Our goal is to identify the main challenges in the study of dental calculus metagenomes across mammalian diversity and to help researchers and collection curators optimise laboratory procedures and plan sampling for future dental calculus studies.

## 2 Materials and Methods

### 2.1 Sample Collection

**FIGURE 1:**
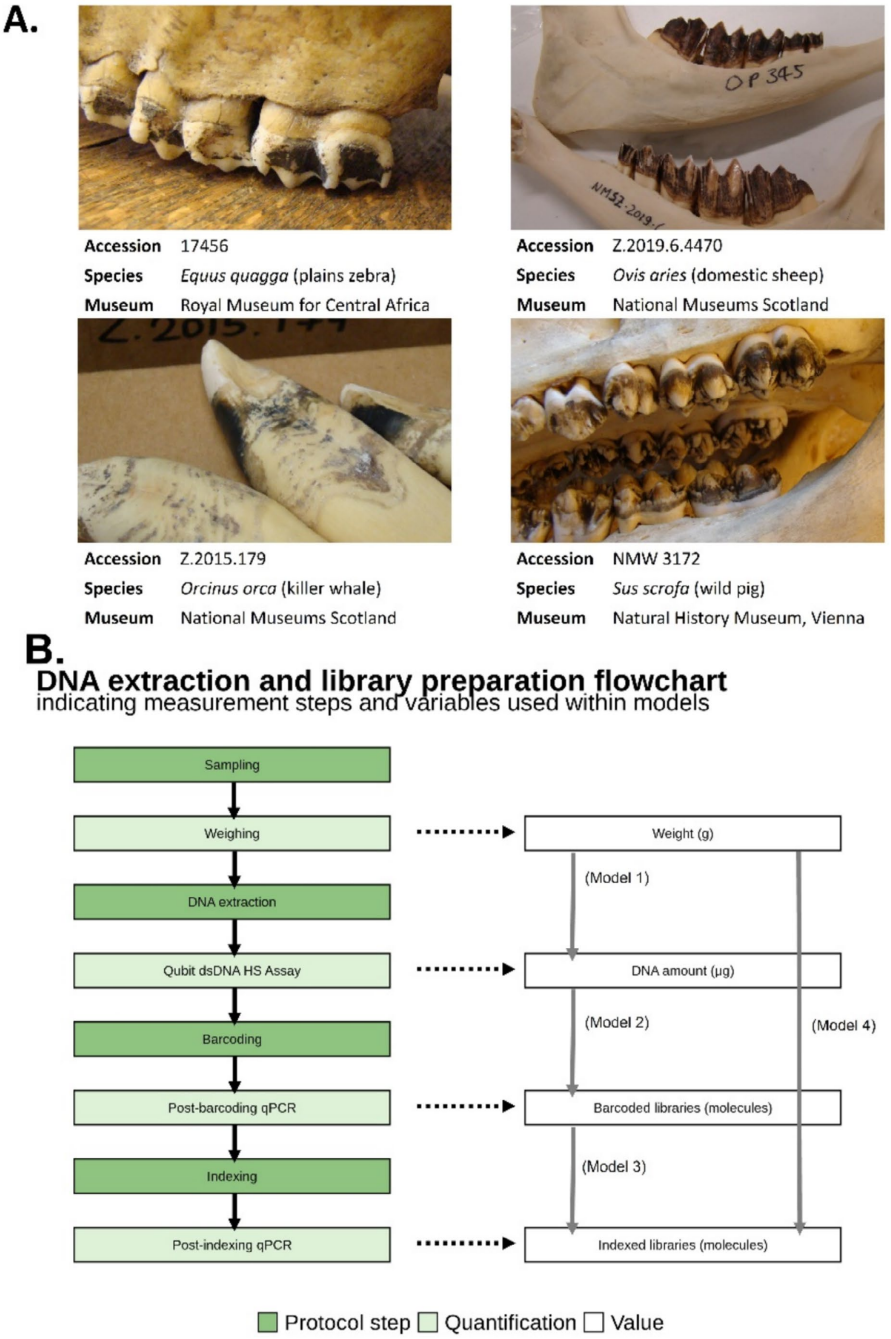
Dental calculus sampling, laboratory and statistical modelling procedures. (A) Top left: An example of ‘chunky’ dental calculus on a plains zebra, *Equus quagga* , specimen housed in the Royal Museum for Central Africa (RMCA). From top right to bottom left, counterclockwise: Examples of film‐like dental calculus on a domestic sheep, *Ovis aries* , in the National Museums Scotland (NMS), a wild pig, *Sus scrofa*, in the Natural History Museum Vienna (NHM Vienna), and an orca, *Orcinus orca* , in NMS. (B) Left: Flowchart representing the laboratory protocol, including DNA extraction and Illumina sequencing library preparation. Dark green indicates protocol steps, whereas light green indicates the quantifications steps. Right: Variables used for the statistical models and tests of this study. Vertical arrows point from the variable used as a predictor (input) to the variable used as a response (output).

Our dataset consists of 515 dental calculus samples belonging to 29 wild mammal species and three domesticates (Table 1), as well as 76 negative controls (50 from DNA extraction and 26 from library preparation, Table S1). The samples were collected from natural history specimens housed in the following institutions: the Natural History Museum Vienna (Austria), the Royal Museum for Central Africa (Belgium), the Natural History Museum London (United Kingdom), the National Museums Scotland (United Kingdom) and the Royal Belgian Institute of Natural Sciences (Belgium). Calculus was collected in a way that minimised contamination during the sampling process, using disposable lab coats, face masks and two layers of gloves, with the upper layer changed before each new specimen. The bench surface on which sampling was carried out was decontaminated with bleach (at 10%) and rinsed with filtered water. Sampling was performed above a sheet of aluminium foil, and calculus was collected onto Whatman Weighing Paper and transferred into 2 mL sterile tubes for storage at room temperature. Dental calculus chunks were dislodged from the teeth by applying pressure at the base of the visible deposit or, if present as a uniform film, gently scraped from the tooth surface using a sterile scalpel blade (Figure 1A), using a fresh blade for each sample. To obtain sufficient amounts of dental calculus for extraction, we often sampled from multiple teeth and/or from both lingual and buccal surfaces of mandibles and maxillaries (Table S1), while avoiding the occlusal surface, which tends to have a distinct microbial composition (Fagernäs et al. 2022). In rare cases, if the amount of material obtained was considered too low, samples from multiple individuals were collected in the same tube, matching individual provenance, sex and age as much as possible (Table S1).

**TABLE 1:** List of species analysed in this study, alongside their taxonomic and dietary categories, and the number of extracted, barcoded and indexed samples. Scientific names are according to the Mammal Diversity Database (2024), with the exception of the Malayan tapir, for which we use the updated name *Acrocodia indica* (Groves and Grubb 2011). The reference genome column includes the NCBI accession of the genome that was used for host mapping, indicating in brackets when the genome taxonomy differed from sample taxonomy, including the reason for the discordance.

Common name	Scientific name	Reference genome	Taxonomic order	Diet category	Samples extracted	Samples barcoded	Samples indexed
African buffalo	*Syncerus caffer* (Sparrman, 1779)	GCA_902825105.1	Artiodactyla	Herbivore	10	10	8
African elephant	*Loxodonta africana* (Blumenbach, 1797)	GCF_000001905.1	Proboscidea	Herbivore	15	13	13
Alpine marmot	*Marmota marmota* (Linnaeus, 1758)	GCF_001458135.2 ( *Marmota marmota marmota* )	Rodentia	Herbivore	13	13	13
Arabian camel	*Camelus dromedarius* Linnaeus, 1758	GCF_036321535.1	Artiodactyla	Herbivore	9	9	9
Argali	*Ovis ammon* (Linnaeus, 1758)	GCA_028583565.1 ( *Ovis ammon polii* [subspecies])	Artiodactyla	Herbivore	11	11	11
Black rhinoceros	*Diceros bicornis* (Linnaeus, 1758)	GCF_020826845.1 ( *Diceros bicornis minor* [subspecies])	Perissodactyla	Herbivore	2	2	2
Bornean orangutan	*Pongo pygmaeus* (Linnaeus, 1760)	GCF_028885625.2	Primates	Frugivore	6	6	4
Central African red colobus	*Piliocolobus foai* (de Pousargues, 1899)	GCA_963574265.1	Primates	Frugivore	26	26	26
Chamois	*Rupicapra rupicapra* (Linnaeus, 1758)	GCA_963981305.1	Artiodactyla	Herbivore	27	27	24
Domestic horse	*Equus caballus* Linnaeus, 1758	GCF_002863925.1	Perissodactyla	Herbivore	6	6	6
Domestic pig	*Sus domesticus* Erxleben, 1777	GCA_017957985.1 ( *Sus scrofa domesticus* [synonym])	Artiodactyla	Frugivore	13	13	13
Domestic sheep	*Ovis aries* Linnaeus, 1758	GCF_016772045.2	Artiodactyla	Herbivore	46	45	42
Dugong	*Dugong dugon* (P. L. S. Müller, 1776)	GCA_030035585.1	Sirenia	Frugivore	5	5	5
Eastern gorilla	*Gorilla beringei* Matschie, 1903	GCA_963575185.1	Primates	Herbivore	9	9	9
Eastern grey kangaroo	*Macropus giganteus* G. K. Shaw, 1790	GCA_028627215.1	Diprotodontia	Herbivore	14	14	14
European badger	*Meles meles* (Linnaeus, 1758)	GCF_922984935.1	Carnivora	Animalivore	39	39	39
European wild pig	*Sus scrofa* Linnaeus, 1758	GCF_000003025.6	Artiodactyla	Frugivore	26	26	26
Giraffe	*Giraffa camelopardalis* (Linnaeus, 1758)	GCA_017591445.1 ( *Giraffa camelopardalis rothschildi* [subpecies])	Artiodactyla	Frugivore	15	15	14
Hippopotamus	*Hippopotamus amphibius* Linnaeus, 1758	GCF_030028045.1 ( *Hippopotamus amphibius kiboko* [subspecies])	Artiodactyla	Herbivore	13	11	11
Indian muntjac	*Muntiacus muntjak* (E. A. W. von Zimmermann, 1780)	GCA_008782695.1	Artiodactyla	Frugivore	13	13	12
Lowland paca	*Cuniculus paca* (Linnaeus, 1766)	GCA_004365215.1	Rodentia	Frugivore	13	12	12
Malayan tapir	*Acrocodia indica* (A. G. Desmarest, 1819)	GCA_031878705.1 ( *Tapirus indicus* [synonym])	Perissodactyla	Frugivore	8	8	7
Muskox	*Ovibos moschatus* (E. A. W. von Zimmermann, 1780)	Not applicable (no samples were sequenced)	Artiodactyla	Herbivore	6	4	0
Okapi	*Okapia johnstoni* (P. L. Sclater, 1901)	GCA_024291935.2	Artiodactyla	Herbivore	14	14	12
Olive baboon	*Papio anubis* (Lesson, 1827)	GCF_008728515.1	Primates	Frugivore	21	21	21
Orca	*Orcinus orca* (Linnaeus, 1758)	GCF_937001465.1	Artiodactyla	Animalivore	4	4	4
Plains zebra	*Equus quagga* P. Boddaert, 1785	GCA_026770645.1 ( *Equus quagga burchellii* [subspecies])	Perissodactyla	Herbivore	27	27	27
Roe deer	*Capreolus capreolus* (Linnaeus, 1758)	GCA_951849835.1	Artiodactyla	Herbivore	49	49	47
South American fur seal	*Arctocephalus australis* (E. A. W. von Zimmermann, 1783)	Not applicable (no samples were sequenced)	Carnivora	Animalivore	1	1	0
South American sea lion	*Otaria flavescens* (G. K. Shaw, 1800)	GCF_009762305.2 ( *Zalophus californianus* [related species])	Carnivora	Animalivore	16	14	14
White rhinoceros	*Ceratotherium simum* (Burchell, 1817)	GCF_000283155.1 ( *Ceratotherium simum simum* [subspecies])	Perissodactyla	Herbivore	13	13	13
Yellow‐backed duiker	*Cephalophus silvicultor* (Afzelius, 1815)	GCA_006410635.1 ( *Cephalophus harveyi* [related species])	Artiodactyla	Frugivore	25	25	25
Total					**515**	**505**	**483**

### 2.2 Dietary Information

In this study, we considered the effect of host diet on the outcome of dental calculus metagenomics. However, since discrete categories such as carnivore, omnivore, etc. are insufficient to describe the dietary diversity among the included mammalian species, we used quantitative estimates for the percentages of different nutrients in the study species' diet, as calculated by Lintulaakso et al. (2023) (Table S2). These included crude protein, crude fibre, ether extract (proxy for fat content), nitrogen‐free extract (proxy for non‐fibrous carbohydrates, such as sugars and starch) and ash (proxy for inorganic compounds). The Lintulaakso et al. (2023) dietary database included all of the study species except for the domestic pig, *Sus domesticus* , so we used the same dietary data as for the European wild pig, *Sus scrofa* . We used the ‘calculated species main diet’ from the Lintulaakso et al. database when discrete dietary categories were needed (Table S2).

**FIGURE 2:**
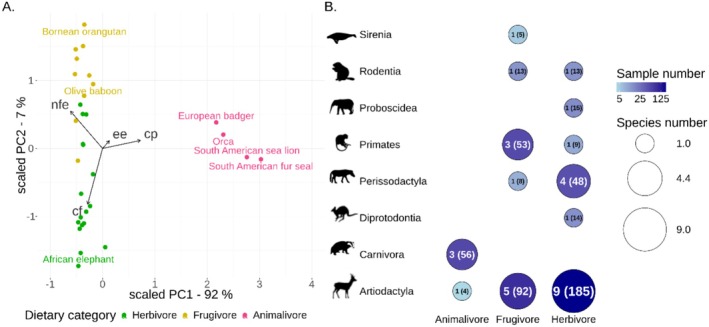
Summary of the dietary and taxonomic diversity in our dataset. (A) Principal component analysis using proximate dietary data for the study species from Lintulaakso et al. (2023) (Table S2), visualised using scaled principal components. The colours represent the dietary category (calculated species main diet) and the arrows indicate an increase in the proportion of specific dietary components: cf = crude fibre; cp = crude protein; ee = ether extract (proxy for fat); nfe = nitrogen‐free extract (proxy for sugars and starches). Species labels are shown for all animalivores and also highlight the olive baboon, *Papio anubis* (which is assigned as ‘undetermined’ in Lintulaakso et al., but was reassigned to frugivore because of its clustering with other frugivores), the African elephant, *Loxodonta africana* (as the species exhibiting the most herbivorous diet along the PC2 axis), and the Bornean orangutan, *Pongo pygmaeus* (as the species exhibiting the most frugivorous diet along the PC2 axis). (B) Summary of the dataset (32 mammalian species, 515 samples), showing the number of species (circle size, number outside brackets) and number of samples (circle colour, number in brackets) for each dietary category and taxonomic order. Animal figures were sourced from http://phylopic.org and are used under Creative Commons licences.

As some nutrients are strongly correlated (e.g., protein and fat are both higher in an animalivorous diet, see Figure S1), we performed a Principal Component Analysis (PCA) (Figure 2A) on the estimated chemical content of the study species diets and extracted the first two principal components, which we scaled and centred on 0 to use as dietary predictors (Table S2). Note that here we use animalivorous as a synonym for carnivorous to distinguish the dietary category from the taxonomic order Carnivora.

### 2.3 Laboratory Protocol and Preparation for Sequencing

The samples were processed in a specialised ancient DNA laboratory with strict anti‐contamination protocols, following the procedure summarised in Figure 1B. DNA was extracted with a silica‐based method developed for ancient DNA (Dabney et al. 2013) and slightly adapted for dental calculus (Brealey et al. 2020). In summary, dental calculus samples were subsampled and transferred to a 2 mL tube, aiming for approximately 25 mg per sample, ranging from 1 mg (in cases where very little dental calculus was available on the specimen) to 35 mg. The samples then underwent surface decontamination with a 10‐min UV irradiation followed by a brief EDTA wash (added 500 μL of 0.5 M EDTA to each tube, mixed by vortexing, incubated for 1 min, and the supernatant was removed following centrifugation at maximum speed for 1 min). Next, the samples were lysed overnight in a buffer consisting of 0.45 M EDTA and 0.025 mg/mL Proteinase K. The next day, the lysate was mixed with 10 mL of binding buffer (5 M GuHCl + 40% (v/v) isopropanol + 0.05% Tween‐20) and 400 μL of 3 M sodium acetate, and the DNA was bound to the silica membrane by centrifugation and washed twice using 450 μL of PE buffer (Qiagen) before being eluted with 45 μL of elution buffer consisting of EB (Qiagen) + 0.05% Tween‐20. Samples were extracted in batches of up to 22, with each batch containing a mix of different host species (Table S1) and two negative controls, one at the start and one at the end of the batch. After elution, the extracts were quantified fluorometrically by a Qubit dsDNA HS Assay (Invitrogen).

Library preparation followed a double‐barcoding double‐indexing protocol to mitigate the effects of index‐hopping (van der Valk et al. 2019). The DNA extract input was 20 μL. However, for some samples that showed very low amplification at the indexing step, suggesting the presence of enzymatic inhibitors, library preparation was repeated after diluting DNA extracts with ultrapure H_2_O in an attempt to ‘dilute out’ the inhibitors. The strength of dilution took into account DNA concentration, ensuring at least 0.02 μL of DNA as input for the library preparation reaction (see Table S1 for DNA input amounts). First, blunt‐ended DNA fragments were created by T4 polymerase. Then, a barcoded adapter was added on each side of the fragment by T4 ligase, and the gaps were filled in by Bst polymerase. We refer to this step as barcoding, otherwise known as adapter ligation (Meyer and Kircher 2010). The resulting barcoded libraries were quantified with qPCR, and those with at least 10^4^ barcoded molecules per μL underwent an indexing PCR using Pfu Turbo Cx Hotstart DNA Polymerase to create complete Illumina libraries, so that each library molecule contained two barcodes and two indices (van der Valk et al. 2019). PCR cycles ranged from 10 to 14, depending on the qPCR results from the previous step, aiming to achieve similar final concentrations for all samples (Table S1). A second qPCR was run to determine the number of fully indexed library molecules, which were then pooled, aiming for equimolar amounts where possible (Table S1). Sequencing was performed on four lanes of the Illumina NovaSeq X Plus platform (PE 150 bp) with a 10% PhiX spike‐in to increase library complexity, aiming for ca. 10 million reads per sample.

### 2.4 Sequence Pre‐Processing and Source Tracking

The sequences were demultiplexed on the basis of their barcode‐index combinations (Brealey et al. 2020), before trimming barcodes and adapters, removing low quality and short (< 30 bp) reads, and merging forward and reverse reads using fastp (v. 0.23.4; Chen et al. 2018). We then mapped the metagenomes onto a combined reference of the human genome (GCA_000001405.29), PhiX (GCA_000819615.1), and the host species (Table S3) using BWA‐aln (v. 0.7.17; Li and Durbin 2009) with options –n 0.04 –k 2 –l 1024 –o 2. Mapped reads were removed using samtools (v. 1.20; Danecek et al. 2021) and unmapped reads, which should be enriched for microbiome‐derived sequences, were retained for downstream analysis. When a host reference genome was not available, we used a related species' genome instead (Table 1, Table S3). To assess whether the proportion of host sequences preserved within dental calculus differs across taxonomically diverse hosts, we used a logistic regression with a quasibinomial distribution, and host order and diet as predictors. We evaluated the effects of these two factors using a Type II analysis of deviance on the logistic regression model, and performed pairwise comparisons across the levels of any significant factors, using generalised linear hypothesis testing implemented in the multcomp R package (v. 1.4.25; Hothorn et al. 2008).

To verify that our data indeed correspond to oral microbiomes, we used decOM (v. 1.0.0; Duitama González et al. 2023), a source tracking tool that partitions metagenomes on the basis of how many k‐mers they share with a set of source environments. Since the default decOM source matrix only includes oral metagenomes from humans, we constructed a custom source matrix which also included oral metagenomes from wild mammals, as well as rumen metagenomes from wild and domesticated animals. More specifically, this source matrix consisted of 478 metagenomes: 177 human oral metagenomes already in the default decOM database, including both ancient (mostly dental calculus) and modern samples (mostly plaque and of undeclared source, as well as saliva, gum and dental calculus); 49 terrestrial mammal oral metagenomes, specifically dental calculus from brown bears and reindeer; 52 marine mammal oral metagenomes, specifically gingival sulcus from dolphins and a harbour seal (49 metabarcoding and three shotgun metagenomic); 40 rumen metagenomes from moose, sheep and cow; 79 sediment/soil metagenomes as well as 81 skin metagenomes from the default decOM database (Table S4).

To identify factors that affect the identifiable source proportions (oral, skin, rumen and soil), we excluded species with less than four samples, combined the different oral categories (human, terrestrial mammals and marine mammals) into one, recalculated the proportions of each metagenome without the ‘Unknown’ partition and then performed a permutational analysis of variance (PERMANOVA), using the ‘adonis2’ function from the vegan package (v. 2.6.6.1; Oksanen et al. 2022) to assess the marginal effects of order, diet and specimen age. We chose to remove the ‘Unknown’ partition to account for reference biases, only considering the proportion of each metagenome that could be characterised. We also specifically assessed if the oral microbiome proportion differs across host order and diet using a logistic regression and generalised linear hypothesis testing, similar to what was described above for the proportion of host DNA.

### 2.5 Evaluating Molecular Protocol Performance Step by Step

#### 2.5.1 Linear Models and Statistical Tests

The output of each step of the protocol (Figure 1B) was modelled using a weighted mixed‐effects model implemented in the lme4 R package (v. 1.1.35.3; Bates et al. 2015) according to the general formula:

$$\begin{array}{cc} \text{Output}\; & \;\sim \;\text{Input}+\text{Input}:\text{dietary}\;\mathrm{PC}1+\text{Input}:\text{dietary}\;\mathrm{PC}2\\ & +\text{Input}:\text{pigmentation}+\left(0+\text{Input}|\text{Species}\right)+\left(0+\text{Input}|\text{Batch}\right) \end{array}$$

where Output and Input are continuous variables that differ for each processing step (i.e., for DNA extraction input was the sample weight in g and output was the DNA amount in μg; see Figure 1B). The predictors dietaryPC1 and dietaryPC2 are the scaled principal components calculated on the basis of the estimated dietary composition of the study species (Figure 2A), with PC1 primarily reflecting the amount of crude protein in the species diet and PC2 distinguishing between crude fibre and sugar/starch. Finally, pigmentation is a logical variable (TRUE/FALSE) indicating visible co‐eluted components in the DNA extract, as detected by eye. Species and Batch indicate the host species and processing batch of the sample, respectively, and were included as random effects on the slope estimate. Specifically, Batch was included as a random effect to account for differences that could be because of technical factors in the laboratory, e.g., variation in buffer concentrations or incubation times. We used weighted least squares because our data were heteroscedastic (showing heterogeneous variance across the range of the response variable), to attribute less weight to observations with high variance.

To account for sample age, an important consideration in historical specimens, we calculated the difference between the dental calculus sampling year and the specimen collection year, and fitted an additional Input:Age interaction term to the general formula described above. Not all specimens had collection year information available and for many samples, only an approximate collection date was recorded (e.g., < 1940 or 1900–1920). The models were hence run on a subset of samples with available collection dates, keeping the most recent year as an approximation for specimens with approximate date (1940 or 1920 respectively for the examples above).

When modelling the indexing step of library preparation, which includes a PCR step, we do not expect a linear relationship between the input (expressed as the number of barcoded library molecules) and output (expressed as the number of indexed library molecules), because the PCR cycles vary between samples. Therefore, we calculated the PCR efficiency E of each reaction on the basis of the following formula (Lalam 2006):

$$N=N_{0}\times {\left(1+E\right)}^{c}$$

therefore

$$E=e^{\ln\;\left(\frac{N}{N_{0}}\right)\times \frac{1}{c}}-1$$

where $N_{0}$ is the initial number of barcoded libraries, N is the final number of barcoded libraries and c is the number of indexing PCR cycles. We then used the same formula with the average E of all reactions to estimate the expected output of the indexing PCR, and we modelled the observed output as a function of the expected output. Similarly to what was described above, we used a subset of samples with available information on the collection year to evaluate the effect of specimen age.

To specifically test for the effect of different dietary categories (herbivore, frugivore and animalivore), we calculated the output to input ratio in each sample (e.g., for DNA extraction, the standardised output reflected the amount of DNA per mg of sample). We then tested how this standardised output differs across dietary categories using Kruskal–Wallis tests. If the global test was significant, we followed up with pairwise Wilcoxon tests, adjusting the *p*‐values using the Holm method.

#### 2.5.2 Machine Learning

As library preparation is a costly and time‐consuming procedure, we wanted to understand if specific sample characteristics are predictive of the probability that a given sample will produce usable Illumina libraries. Such relationships may depend on non‐monotonic interactions. For instance, there is likely an optimal DNA concentration for library preparation, with efficiency declining at both very low and very high input amounts, and this optimum may differ between different sample types: for instance, if inhibitors are present, the optimum may be at a lower sample input. To identify such patterns, we performed binary classification in an attempt to predict the probability of success of the indexing protocol, using a machine learning approach implemented in R using the mlr3 package (v. 0.20.2; Lang et al. 2019). We defined indexing failure when fewer than 10^9^ indexed molecules (in 20 μL reaction volume) were produced, which corresponds to approximately the first quartile of indexed fragments for all negative controls (1.2 × 10^9^; Figure S2A, Table S1). As predictors, we used DNA input (in μg), barcoded library volume used (μl), presence of extract pigmentation, host order and diet (expressed in scaled dietary PC1 and PC2; Figure 2A). We first split our samples into a training (*N* = 324) and a testing dataset (*N* = 159), aiming for similar distributions of host species and number of coloured extracts in both sets (Table S1). We tested learning algorithms (‘learners’) on the basis of different approaches: k‐nearest neighbours (‘kknn’), single layer neural network (‘nnet’), random forest (‘ranger') and classification tree (‘rpart’) (Lang et al. 2024). We first optimised (‘tuned’) these learners by testing various hyperparameters using a Bayesian optimisation tuner (Schneider et al. 2024) and evaluating them using repeated cross‐validation (10 folds, 10 repeats) within the training dataset. After we identified the hyperparameter combinations that, for each learner, achieved the best predictions without overfitting, we evaluated the predictions of these tuned learners on the test dataset using the log loss metric. A featureless learner, which always predicts the majority class from the data it is trained on (in our case, this was indexing success), was used to inform the minimum acceptable performance for the other learners. More details on the hyperparameters tested can be found in Table S5. We finally used the best performing model, generated with a ‘ranger’ algorithm (an implementation of the random forest method) to estimate the relative importance of the predictor variables. We also generated predictions for indexing success using hypothetical samples—a host type and sample input that was not always present in our data. These consisted of DNA input amounts ranging from 0 to 0.5 μg (using brackets of 0.05 μg) for samples representing three types of hosts: members of the order Carnivora with animalivorous diets (scaled PC1 = 2.5, scaled PC2 = 0, see Figure 2A and ‘Dietary information’ section above), Artiodactyla with herbivorous diets (scaled PC1 = −0.5, scaled PC2 = −1) and Primates with frugivorous diets (PC1 = −0.5, PC2 = 1).

## 3 Results

### 3.1 Dataset Characteristics

Our dataset consists of 515 samples from 32 mammalian species (Table S1) with 1 to 499 samples per species (median = 13), representing eight taxonomic orders and a diversity of diets (Figure 2B, Table 1). As expected, most taxonomic orders have representatives from only one or two dietary categories, but our selection of taxa to sample was designed to limit intercorrelation of the two factors as much as possible.

A PCA on the basis of the dietary composition of the study species (Table S2) revealed two main clusters along the first principal component (PC1, Figure 2A). One cluster was characterised by high protein content and contained the European badger ( *Meles meles* ) and three marine animalivores. The second cluster contained herbivores and frugivores. The second principal component (PC2) distinguished herbivores with high fibre diets from frugivores with higher sugar/starch diets. On the basis of these groupings, we have amended the dietary classification for the olive baboon ( *Papio anubis* ) in our dataset, which was reported as ‘undetermined’ in Lintulaakso et al. (2023), to ‘frugivore’.

We observed the presence of pigmented DNA extracts (usually with a brown or yellow hue) during sample processing, indicating the presence of secondary compounds co‐eluting with DNA. Pigmented extracts were more common among herbivores (29.9%) and frugivores (19.9%) than in animalivores (1.6%) (Figure S2B). The species with the largest proportion of pigmented extracts were the plains zebra ( *Equus quagga* ; 19 out of 27 samples), the muskox ( *Ovibos moschatus* ; 4 out of 6), the Indian muntjac ( *Muntiacus muntjak* ; 7 out of 13), the giraffe ( *Giraffa camelopardalis* ; 8 out of 15), the okapi ( *Okapia johnstoni* ; 7 out of 14) and the Malayan tapir ( *Acrocodia indica* ; 4 out of 8). Secondary compounds that produce pigmented DNA extracts can often act as inhibitors for enzymatic reactions such as PCR; therefore, this factor was included as a predictor when modelling the outcome of laboratory protocols (see Models 1, 2, 3 and 4 below).

### 3.2 Host Diet Affects DNA Concentration of Dental Calculus Extracts

We explored the relationship between the weight of dental calculus used for DNA extraction and the resulting amount of DNA in the eluate, while accounting for the potential influence of host diet and extract pigmentation (included as interaction terms with weight) using a mixed linear model with weighted least squares (Model 1), after excluding one outlier sample with DNA output > 4 μg.

Model 1

$$\begin{array}{cc} \text{DNAout}\; & \;\sim \;\text{Weight}+\text{Weight}:\mathrm{PC}1+\text{Weight}:\mathrm{PC}2\\ & +\text{Weight}:\text{Pigmentation}+\left(0+\text{Weight}|\text{Species}\right)\\ & +\left(0+\text{Weight}|\text{extractionBatch}\right) \end{array}$$

where DNAout refers to the extracted DNA amount (in μg) and Weight refers to sample weight used as input. Although the model was fitted using sample weight in g, for interpretability, we report weight mg in the main text and figures and have rescaled the coefficients accordingly. The original coefficients are found Table S6. As our model did not fit the assumptions of normality of variance (Shapiro–Wilk normality test: *W* = 0.85, *p* < 0.001, Figure S3), we used weighted least squares instead of ordinary least squares. We also investigated our model for multicollinearity by calculating variance inflation factors (VIF) and found no evidence for correlation between variables (VIF < 1.15; Table S6).

**FIGURE 3:**
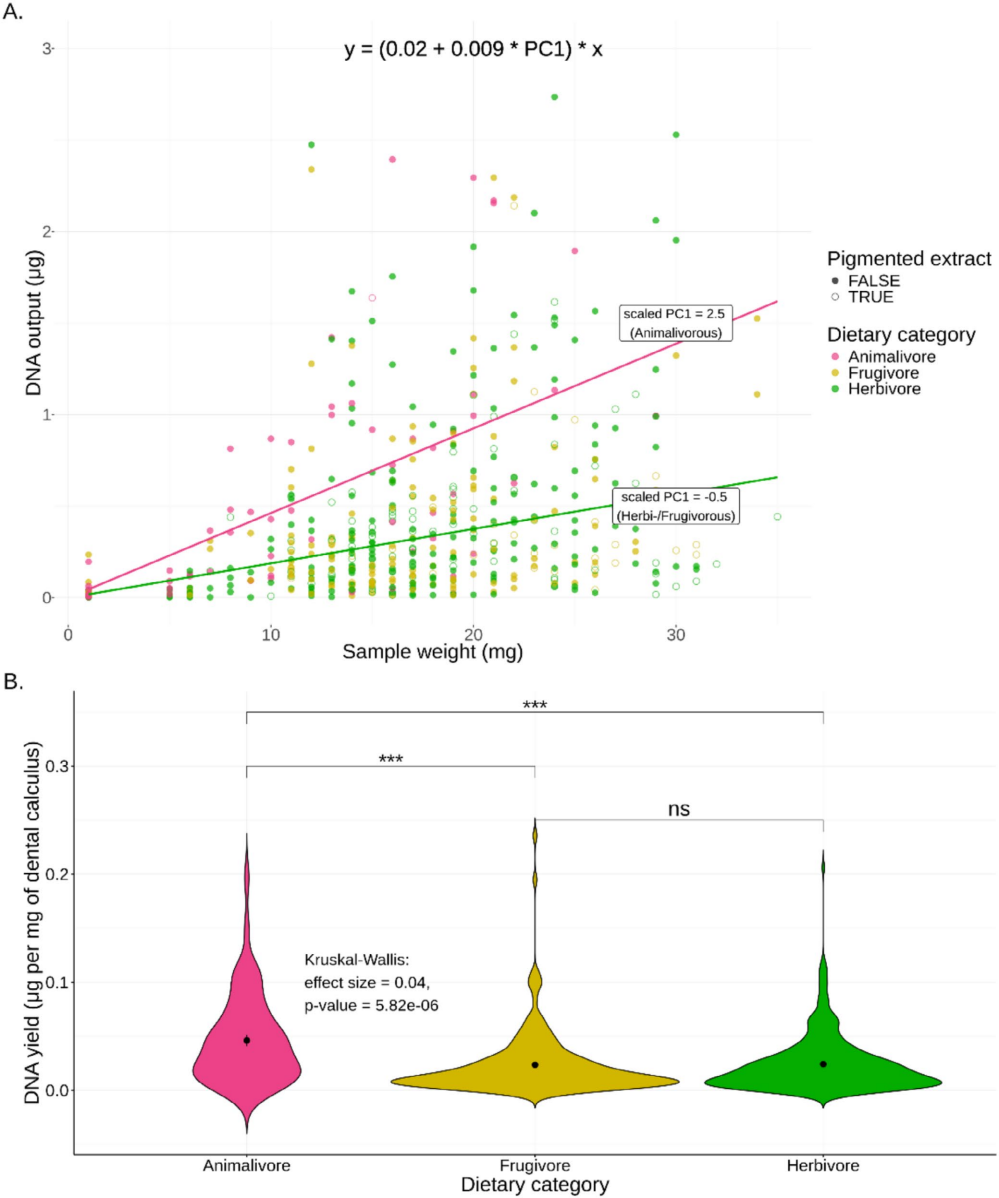
Factors affecting DNA output following dental calculus DNA extraction. (A) The relationship between sample weight (in mg) and extracted DNA (in μg) for 515 samples. The formula on the top is based on all significant model estimators and the two lines show the expected DNA concentration for a species with scaled PC1 = 2.5 (indicative of an animalivorous diet) and a species with a scaled PC2 = −0.5 (non‐animalivorous diet). (B) Normalised DNA yield (in μg of DNA per mg of sample) for different dietary categories. Significance levels:****p < 0.0001, (np) *p* > 0.05.

As expected, the model showed that sample weight had a positive effect on extracted DNA amount (*p* < 0.001), suggesting that for every mg of dental calculus sampled we extract approximately 0.023 μg of DNA (Figure 3A, Table S6). We also found a positive interaction with dietary PC1 (which reflects the animalivory levels) with an interaction coefficient of 0.009 (*p* = 0.01). This suggests that we retrieve more DNA per mg of sample for more animalivorous host species. According to our estimates, we can expect 0.046 μg of DNA per mg of dental calculus for a typical animalivore (with PC1 = 2.5, see Figure 3A), but only 0.018 μg per mg of sample for a herbivore (PC1 = −0.5) (Figure 3A; Table S6).

Considering a subset of 495 samples for which the collection year was available, we identified, in addition, a significant negative effect of specimen age on DNA amount, suggesting that a specimen loses approximately 0.009 μg of DNA per mg of dental calculus per 100 years (Table S6). This is consistent with the expectation that older samples tend to have more degraded DNA, although DNA fragmentation is not a strictly linear process (Sawyer et al. 2012). The contribution of sample weight and dietary PC1 to the model was not affected by the inclusion of specimen age, and there was no evidence for multicollinearity according to the calculated VIFs (Table S6).

We also tested for differences in DNA yield (expressed as proportion of sample weight) between discrete dietary categories (Figure 3B). Indeed, diet category had a significant effect on the DNA yield (Kruskal–Wallis test: *p* < 0.001, effect size = 0.04; Figure 3B), with animalivore hosts having more DNA per mg of extracted dental calculus, confirming the above results.

### 3.3 Pigmented Dental Calculus Extracts Negatively Affect Library Preparation

**FIGURE 4:**
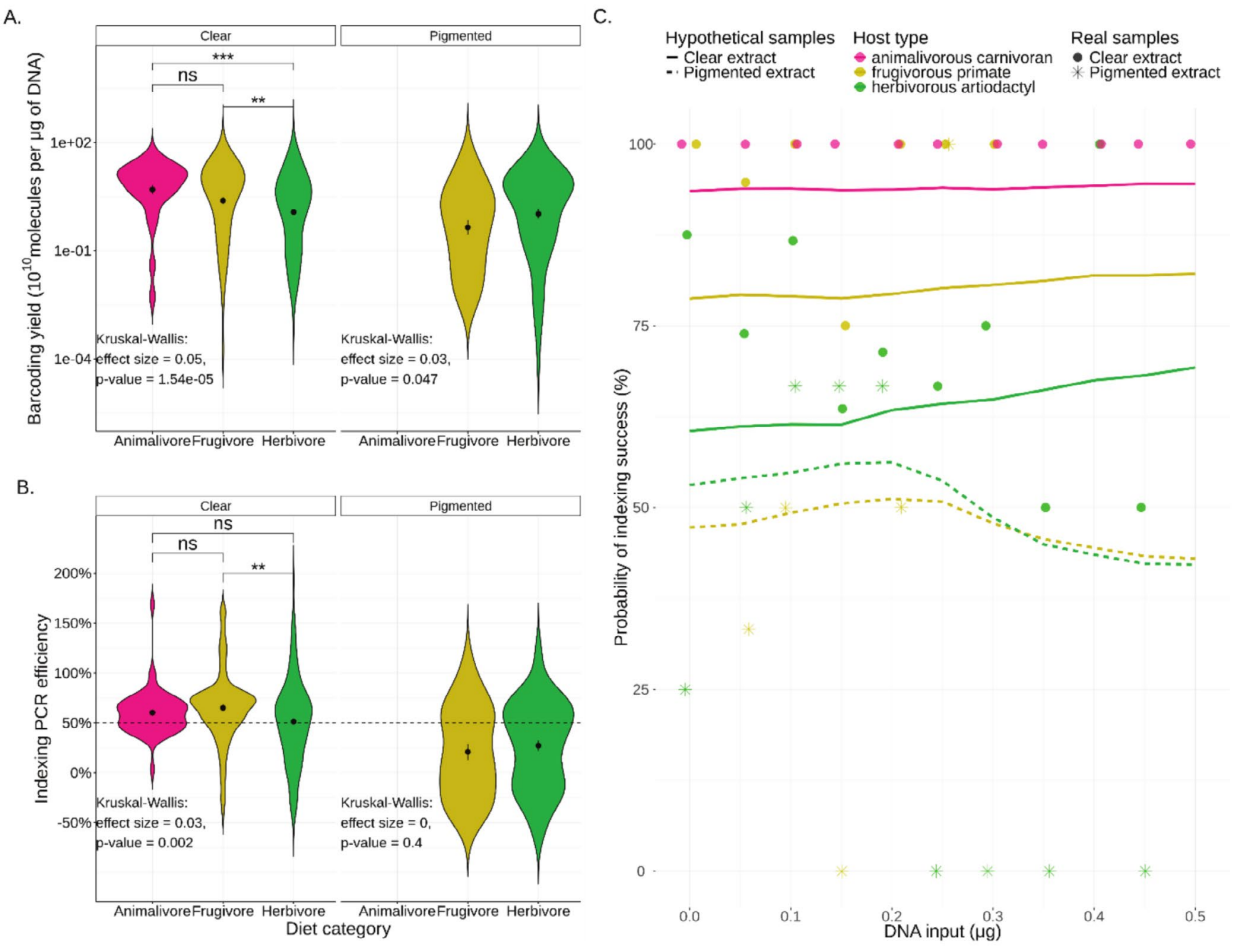
Outcome of library preparation by dietary category and extract pigmentation. (A) The amount of barcoded libraries (in 10^10^ molecules) per μg of input DNA. Note the logarithmic scale of the y‐axis. (B) The estimated indexing PCR efficiency per sample. Under ideal conditions the efficiency would be 100%, indicating a doubling of DNA fragments at each cycle. The horizontal dashed line indicates the average efficiency (49.9%) across all indexed samples in our dataset. The effect of diet category was assessed using Kruskal–Wallis tests separately for clear and pigmented extracts, pairwise comparisons were performed with Wilcoxon tests and *p*‐values were adjusted using the Holm method. Significance levels: ***p < 0.001, **p < 0.01, (ns) *p* > 0.05. (C) Indexing success probability (displayed as lines) as predicted by a ranger classifier trained on a subset of our data. The predictions were generated for hypothetical samples with DNA input spanning every 0.05 μg from < 0.01 to 0.5 μg, with clear or pigmented extracts, and with features typical of three host types present in our dataset: Animalivorous carnivorans (scaled PC1 = 2.5, scaled PC2 = 0, pink lines), herbivorous artiodactyls (scaled PC1 = −0.5, scaled PC2 = −1, green lines) and frugivorous primates (scaled PC1 = 0.5, scaled PC2 = 1, yellow lines). The coloured circles show observed indexing success values of similar hosts: Animalivorous carnivorans represented by the badger and the South American sea lion (48 samples), herbivorous artiodactyls represented by the Arabian camel, the roe deer, the hippopotamus, the okapi, the argali, the domestic sheep, the chamois and the African buffalo (146 samples) and frugivorous primates represented by the olive baboon, the Central African red colobus and the orangutan (50 samples). The real indexing success was calculated within bins of DNA input μg that approximated the values used for the hypothetical data (see Table S7).

The 505 samples that were retained after DNA extraction underwent barcoding, during which a double‐sided barcode was added to each DNA fragment. We modelled this first library preparation step (barcoding) as a linear mixed model with weighted least squares (to account for heteroscedasticity; Figures S4 and S5), similar to the formula used to model DNA extraction. Specifically for the barcoding step, we first excluded seven outliers with barcoding outputs larger than 8 × 10^10^ copies and one with a DNA input amount larger than 3 μg, and used the following formula:

Model 2

$$\begin{array}{cc} \text{barcodedout}\; & \;\sim \;\text{DNAin}+\text{DNAin}:\mathrm{PC}1+\text{DNAin}:\mathrm{PC}2\\ & +\text{DNAin}:\text{pigmentation}+\left(0+\text{DNAin}|\text{Species}\right)\\ & +\left(0+\text{DNAin}|\text{barcodingBatch}\right) \end{array}$$

where barcodedout is the output of barcoded libraries expressed in 10^10^ molecules and DNAin is the input DNA in μg. We observed no multicollinearity in this model (VIFs < 1.04; Table S6). As expected, we found a positive relationship between DNA input and barcoding output with a slope of 2.8 (*p*‐value < 0.001), but this relationship was affected by extract pigmentation with an interaction of −2.2 (p‐value = 0.004) (Table S6), suggesting that, when dealing with pigmented extracts, increasing the DNA input amount may only have marginal or no benefits for the barcoding output (Figure S4A, Table S6). We also found a marginal effect of the dietary PC2 (frugivory versus herbivory axis) suggesting that herbivores may be generating a lower barcoding output than frugivores (Table S6). This became more evident when discrete dietary categories were compared. Clear DNA extracts from herbivores had a lower barcoding output than extracts from hosts with other diets (pairwise Wilcoxon tests: *p* < 0.01, Figure 4A). We identified no significant effect of specimen age on barcoding output, when investigating a subset of samples (*N* = 479) for which this information was available (Table S6).

After barcoding, 22 libraries were excluded because of too low numbers of library molecules (less than 100× barcoded molecules compared to the negative control in that library batch). Of these, 63.6% (*n* = 14) had a pigmented extract, whereas only 21.8% (*n* = 104) of the retained 475 barcoded libraries did, suggesting that extract pigmentation may reflect the presence of enzymatic inhibitors, which impede the barcoding step.

The retained libraries underwent an indexing PCR, with the number of PCR cycles adjusted according to the template concentration (Table S1). We found that a considerable proportion of the dataset failed to amplify as expected, with 100 (20.7%) of indexed libraries producing fewer copies than most negative controls, which contained no sample and started at a much lower concentration. Since controls produced a median of 4 × 10^9^ indexed copies (range: 3 × 10^8^–1 × 10^12^) (in 20 μL reaction volume), we considered samples with less than 10^9^ copies to have failed indexing (Table S1). Of these failed libraries, 47% (*n* = 47) had a pigmented extract, whereas of the successful libraries (> 10^9^ indexed copies), only 15.1% (*n* = 58) did (Figure S2B).

We estimated the efficiency of the indexing PCR to be on average 49.9% (reflecting an increase of DNA molecules by 149.9% at every cycle). However, we found that pigmented extracts showed a lower PCR efficiency (mean: 24.8%) than clear extracts (mean: 57.8%, Kruskal–Wallis test: *p* < 0.001, effect size = 0.08; also see Figure 4B, Figure S5B). Even among clear extracts, herbivores tended to have somewhat reduced efficiency (Figure 4B), similar to what we observed at the barcoding step. To better understand the relationship between the observed (indexout) and the expected (exp) indexed molecules (under the assumption that all samples have an average PCR efficiency of 49.9%), we implemented a linear mixed effects model (Model 3) with weighted least squares (once again to account for heteroscedasticity in the data; Figure S6), after removing three outliers with more than 400 × 10^10^ observed indexed molecules.

Model 3

$$\begin{array}{cc} \text{indexout}\; & \;\sim \;\exp+\exp:\mathrm{PC}1+\exp:\mathrm{PC}2+\exp:\text{pigmentation}\\ & +\left(0+\exp|\text{Species}\right)+\left(0+\exp|\text{indexingBatch}\right) \end{array}$$

Again, we found no evidence of multicollinearity (VIFs < 1.14; Table S6). We observed a positive relationship between the observed and expected indexing with a coefficient of 1.50 (*p* = 0.002), very close to the average PCR efficiency, which is expected given that we used PCR efficiency to calculate the expected indexing output. However, we found a negative effect of pigmentation equal to −0.67 (*p* = 0.002, Figure S5B, Table S6). We did not detect an effect of diet as expressed by dietary principal components nor specimen age (Table S6).

At the end of the entire protocol, 383 successfully indexed libraries were obtained from the initial 515 extracted samples, indicating an overall success rate of 74.4%. To assess the likely success of the laboratory methods as early as possible during sample processing and to derive general recommendations from the moment of sample collection, we ran a weighted mixed effects model to determine the impact of sample input weight on the final indexing output:

Model 4

$$\begin{array}{cc} \text{indexout}\; & \;\sim \;\text{Weight}+\text{Weight}:\mathrm{PC}1+\text{Weight}:\mathrm{PC}2\\ & +\text{Weight}:\text{Pigmentation}\\ & +\left(0+\text{Weight}|\text{Species}\right) \end{array}$$

We found a small positive effect of sample weight, suggesting that the indexing output increases by 1.49 × 10^10^ indexed molecules for each μg of sample (the average output is 42.38 × 10^10^), as well as a negative effect of extract pigmentation (Table S6). In addition, using a subset of 455 samples with information on collection year, we found an overall negative effect of specimen age (Table S6).

Although our linear mixed models suggest that at each protocol step input has a positive impact on the output, it is possible that this relationship is nonlinear, or non‐monotonic, as there may be an optimal amount of DNA input, which can vary depending on the presence of enzymatic inhibitors. To investigate such nonlinear relationships, and in the interest of planning future research, we employed a machine learning method to model the success probability of the indexing step, using information that is available at the DNA extraction stage. Specifically, given the high failure rate at the indexing step (*N* = 100, or 20.7% with less than 10^9^ molecules; Figure S2A), we performed binary classification using as predictors the following variables: DNA input (μg), host order, host diet expressed quantitatively as scaled PC1 (animalivory axis) and PC2 (herbivory vs. frugivory axis), presence/absence of extract pigmentation and barcode library volume (μL). The minority class (indexing failure) was coded as TRUE, and the majority class (indexing success) was coded as FALSE. The best performing learner was the ranger algorithm (with hyperparameters mtry = 3, num.trees = 985, min.node.size = 20, max.depth = 18; Table S5). However, even this best‐performing learner had a relatively small predictive power, with an Out‐of‐Bag error of 0.12, a moderate false‐negative rate (FNR = 0.02), but a rather high false‐positive rate (FPR = 0.85). The high FPR shows that the model predicts some successful samples to fail indexing, and this may be partly due to the relatively low number of true positives in our dataset (21% of samples). The resulting model suggests that the most important factors determining indexing output are, in descending order, extract pigmentation, dietary PC2 (herbivory vs. frugivory axis), dietary PC1 (the animalivory axis), barcoded library input, host taxonomic order and finally the DNA input (Table 2).

**TABLE 2:** Variable importance for indexing success/failure, as estimated using a ‘ranger’ (random forest) machine learning algorithm.

Variable	Importance
Extract pigmentation	8.57
Dietary PC2	6.58
Dietary PC1	5.76
Barcoded library volume (μl)	5.14
Host taxonomic order	4.34
DNA input (μg)	3.15

To better understand how DNA input impacts the success rate of library preparation for different types of samples (in terms of extract pigmentation, host taxonomic order and host diet), we used the trained model to make predictions for hypothetical samples. We provided values representative of the three different dietary categories, with DNA input ranging from < 0.01 to 0.5 μg and with clear and pigmented extracts (Table S7). The ranger model agreed with the linear models (Models 2 and 3) in that extract pigmentation and herbivory adversely affect library preparation (Figure 4C). Indexing success for pigmented and/or herbivorous dental calculus samples was predicted to be between 42.1% and 69.3%, whereas for clear extracts from animalivorous or frugivorous species, it was between 78.7% and 94.5%. The additional contribution of the machine learning model, beyond the insights of the linear model, is an indication that samples with pigmented extracts start performing worse when input increases past 0.2 μg of DNA (Figure 4C, Table S7). However, the model does not capture the decrease in the success rate of non‐pigmented herbivore samples, predicting instead a consistently low success rate (Figure 4C). The decreasing success rate of pigmented extracts (and to some extent herbivorous hosts) may reflect inhibitor content increasing with higher sample inputs.

### 3.4 DNA Input Has Low Impact on Library Complexity

In total, 401 indexed dental calculus metagenomic libraries were sequenced on an Illumina NovaSeq platform. After pre‐processing the metagenomic reads, each sample had on average 6.9 million reads (and up to 52.6 million, Table S3). So far, we have explored how the amount of DNA used for library preparation affects its efficiency and success rate. However, a concern in metagenomics, especially when working with degraded ancient or historical samples, is the information content of a sample (often referred to as library complexity), which is related to the diversity of DNA fragments. In this sense, DNA input can be viewed as ‘s ampling effort’, with sequencing libraries generated from low‐input DNA showing fewer unique library molecules and hence lower complexity.

**FIGURE 5:**
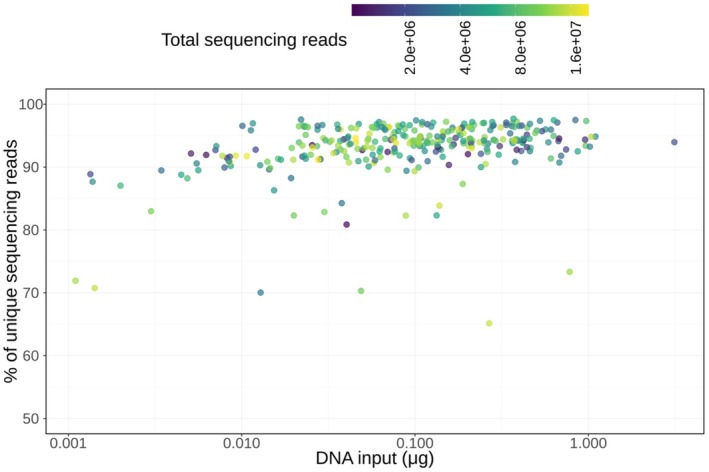
Percentage of unique sequencing reads (after adapter and barcode trimming) plotted against the DNA input (in μg) used for library preparation. The colour indicates the total sequencing depth for each sample. For this visualisation, we excluded samples with fewer than 10^6^ or more than 2 × 10^7^ reads, retaining 302 samples. Note the log10‐transformed *x*‐axis and colour scale.

To test this hypothesis, we investigated how DNA input affects the percentage of unique sequenced reads (after trimming adapters and barcodes). We considered a subset of 302 samples that achieved a sequencing depth between 10^6^ and 2 × 10^7^ reads. The percentage of unique reads reaches a plateau at around 95% once DNA input reaches 0.01 μg (Figure 5). Sequencing depth can also impact the percentage of unique reads (even with low input, a very shallowly sequenced library will have mostly unique reads), and since our samples varied considerably in sequencing depth, we also inspected subsets of samples with similar sequencing depths. We split our data into four subsets, each reflecting a quartile of sequencing depth (Figure S7) and found that library complexity plateaus much faster for sequencing depths below 10 million reads. Overall, for DNA input > 0.01 μg, 92.5% of samples (258 out of 279 samples) showed > 90% unique library molecules. For samples with DNA input < 0.01 μg, only 52.2% of samples (12 out of 23) reached this library complexity. It is important to consider that the sequencing of this study is shallow by many metagenomics standards, and therefore, for more deeply sequenced data, higher DNA input may be necessary to maintain sufficiently complex libraries for downstream analysis.

Nevertheless, this analysis demonstrates that the DNA input amount considered as ideal for successful library preparation (0.2 μg; Figure 4C) is sufficient to obtain sequencing libraries with good complexity. Considering the estimated average yield of 0.023 μg (23 ng) of DNA per mg of dental calculus, this DNA amount corresponds to approximately 8.69 mg of sample.

### 3.5 Host Genomic and Oral Microbiome Content Vary Across Mammals

After excluding the human‐mapped sequences as contamination, we calculated the percentage of host reads found in the dental calculus metagenomes, which was on average 6.3% (range: 0% to 83.7%; Figure S8). The analysis of deviance suggested a significant effect of host order (Type II analysis of deviance: chi‐squared = 19.0, *p*‐value = 0.008), but not host diet (chi‐squared = 1.9, *p*‐value = 0.39) or specimen age (chi‐squared = 19.3, *p*‐value = 0.86) in determining the proportion of host reads. The host diet exhibited evidence for moderate multicollinearity (adjusted GVIF = 2.5), which warrants some caution in interpreting the results, but is not concerning. Pairwise comparisons between host orders showed that African elephants (order Proboscidea) generally had higher host content (1.2%–81%, median 5.3%; Figures S8 and S9) than most other hosts (Artiodactyla, Perissodactyla and Primates) (Table S8, Figure S9). Sirenia had consistently the lowest host content (0.24%–0.77%, median 0.57%; Figures S8 and S9), but were not significantly different from other host orders, likely due to their small sample size (*N* = 5). Primates also tended to have fewer host reads (Figures S8 and S9). These results may reflect technical artefacts rather than differences in the intrinsic properties of dental calculus (see Section 4).

**FIGURE 6:**
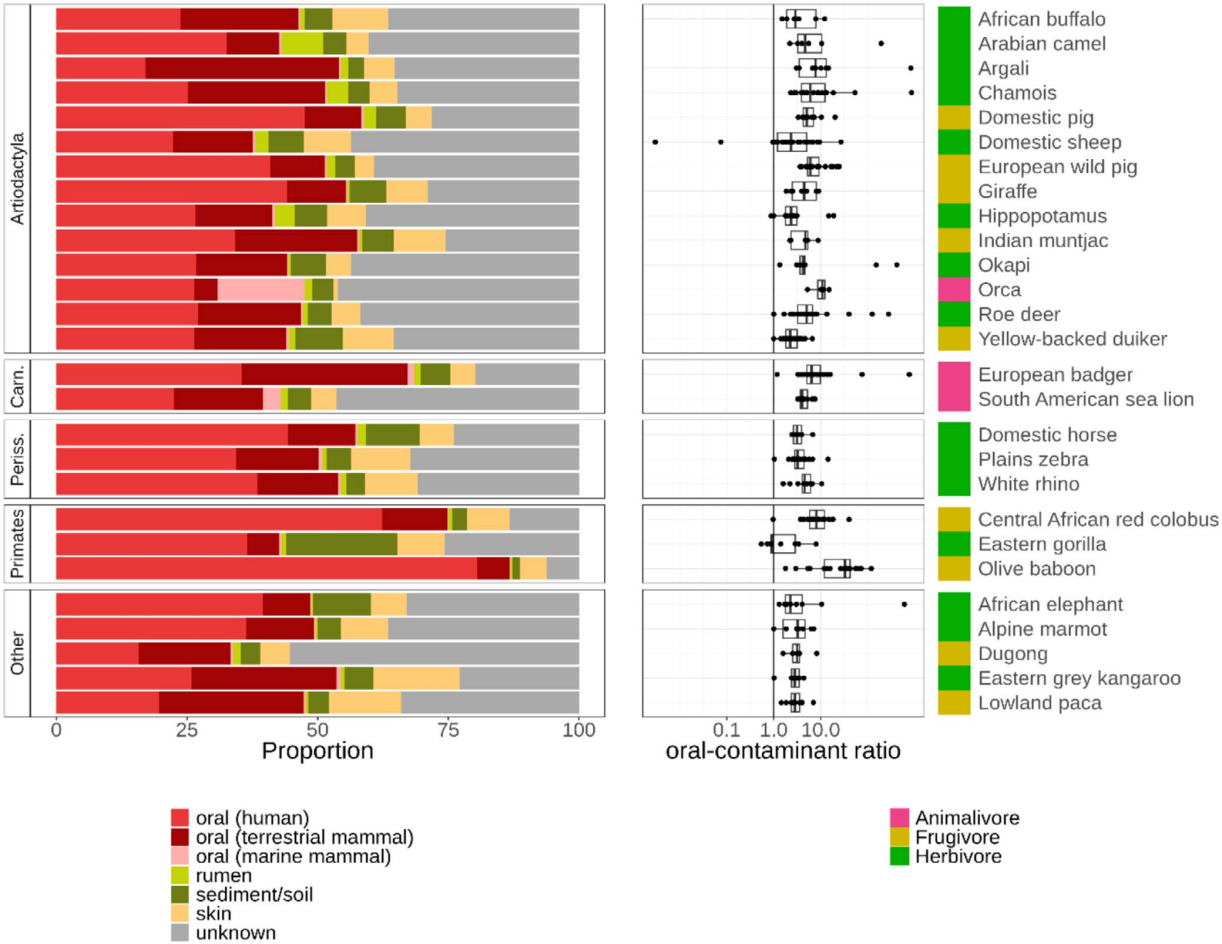
Source tracking of the dental calculus metagenomes. Left: Estimated source composition of dental calculus metagenomes (after removing reads mapping to the host and human genome) averaged across samples for each host species. Analysis on the basis of 383 samples from 27 species with at least five samples each (see representation per sample in Figure S10). The ‘Unknown’ partition refers to k‐mers not represented in the included sources. Plot data can be found in Table S3. Right: Per‐sample oral to contaminant ratio per species (where oral includes human, terrestrial and marine mammal, and contaminant includes skin and soil). The vertical line indicates a ratio of 1. Note the logarithmic scale on the *x*‐axis. The coloured bar on the right‐hand side indicates the mammalian host diet.

After removing host and human reads, we estimated the oral microbiome proportion among the remaining sequencing reads. To this end, we used decOM for 27 species with at least five samples each (383 samples in total), confirming the presence of the oral microbiome signature in all host species (Table S3, Figure S10). On average, 53.7% of the metagenomes were estimated to resemble the oral microbiome (human, terrestrial mammal and marine mammal considered together). Skin and soil/sediment microbiota, which are likely contaminants (although soil could be co‐ingested during feeding), accounted for an average of 7.2% and 5.5%, respectively, whereas rumen amounted to 1.4% (Figure 6, Table S3, Figure S10). On average, 32.1% of dental calculus metagenomes were of unknown origin (i.e., not matching any of the included sources, Figure 6). The high unknown proportion is expected in wild animals with uncharacterised microbiomes. The two host species with the largest metagenomic proportion of unknown source were both marine mammals, the dugong ( *Dugong dugon* : on average 55.4% unknown) and the South American sea lion ( *Otaria flavescens* : 46.4% unknown, Figure 6). These differences may not really reflect the preservation of the oral microbiome in dental calculus, but instead our ability to characterise it (see Section 4).

Disregarding the unknown fraction, we find that both order and dietary category had small and significant effects on the source metagenome composition of dental calculus metagenomes (PERMANOVA marginal effects: order *R* ^2^ = 0.04, *p*‐value = 0.037; dietary category *R* ^2^ = 0.02, *p*‐value = 0.02). More specifically, the oral microbiome proportion varied between taxonomic orders (Type II analysis of deviance: chi‐squared = 909.0, *p*‐value < 0.001) and to a lesser extent by diet (chi‐squared = 34.0, *p*‐value < 0.001) and specimen age (chi‐squared = 7.6, *p*‐value = 0.006). Again, the host diet showed somewhat high, but not concerning, multicollinearity (adjusted GVIF = 1.9). Oral microbiome proportion was greater than the proportion of likely contaminants (skin and sediment/soil microbiota combined) in most samples and host species (Figure 6). We found that Primates had a higher oral microbiome proportion than all other host orders (Generalised linear hypothesis testing: 1.5 ≤ ratio ≤ 6.1, *p*‐value < 0.05) (Table S9, Figure 6, Figure S10). On the other hand, dugongs ( *Dugong dugon* ), the only representative of the order Sirenia, had the lowest oral microbiome proportion (0.16 ≤ ratio ≤ 0.46; *p*‐value < 0.001), followed by Rodentia (Table S9).

## 4 Discussion

Dental calculus is an untapped resource for microbiome studies across a diversity of mammals (Brealey et al. 2020; Moraitou et al. 2022; Ottoni et al. 2019; Fellows Yates et al. 2021). With growing interest in this field, it is crucial to devise optimal processing methods. Until now, metagenomic analyses of dental calculus have been carried out in only a few mammalian species, with a narrow representation of ecological (e.g., diet, lifestyle) and taxonomic diversity. In this study, we massively increase this diversity by analysing dental calculus from 32 species across the mammalian tree of life, including marine, aquatic and terrestrial taxa with representatives from eight extant mammalian orders. We recovered an oral microbiome signature from all studied species, confirming the utility of dental calculus for oral microbiome investigations in many previously unstudied species, but also found that not all host species perform equally well in the laboratory.

The most striking, albeit not unexpected result, is the severely detrimental effect of DNA extract pigmentation on the efficacy of the molecular laboratory protocols, particularly the library preparation steps. Pigmented extracts produced fewer barcoded and indexed molecules (Figure 4) and were removed from the experiment at a higher rate because of this lower performance. DNA extract colouration has been linked to the presence of secondary compounds that negatively impact the enzymatic steps of the molecular laboratory workflow, particularly pectins, polyphenols, polysaccharides and humic acid (Sidstedt et al. 2015, 2020; Loomis 1974). Plants are particularly rich in enzymatic inhibitors (Schrader et al. 2012; Demeke and Adams 1992; Winkel‐Shirley 2001), which may explain why herbivores and frugivores showed a higher proportion of pigmented DNA extracts than animalivores (Figure S2B). Tropical and subtropical plants are especially enriched in compounds such as phenols to defend themselves against herbivory (Becerra 2015) and, correspondingly, the species with the largest proportion of pigmented extracts in our dataset (plains zebra, muntjac, giraffe, okapi, malayan tapir and muskox) were almost exclusively tropical/subtropical browsers and/or grazers (with the exception of the muskox). Therefore, it seems that enzymatic inhibition in dental calculus samples may be caused by a diet rich in secondary compounds. Even without evident pigmentation, a herbivorous diet had a negative effect on the efficacy of the barcoding and indexing steps (Figure 4, Figure S4). As extract pigmentation was recorded by visual inspection, low concentrations of inhibitors may have remained undetected, but were likely present in herbivorous hosts.

The presence of enzymatic inhibitors poses challenges not only in studies of dental calculus but also in other historical and ancient samples, including sediments (Nota et al. 2022; Rayo et al. 2022). Sample dilution, which is often employed to ‘dilute out’ the inhibition, can be prohibitive for very degraded, low‐quantity material. Our findings suggest that this may be a good practice for samples with a high inhibitor load: pigmented extracts have a higher predicted success rate for DNA inputs below 0.2 μg (Figure 4C, Table S7). In addition, we retrieved > 90% unique reads for most libraries with at least 0.01 μg of DNA input (Figure 5, Figure S7). In conjunction, these findings suggest that diluting to a final concentration between 0.01 and 0.2 μg (10–200 ng) may help evade inhibition without sacrificing library complexity. Assuming the average DNA yield of 0.023 μg per mg of dental calculus (on the basis of Model 1), this indicates that using more than 8–9 mg may increase the chance of inhibition. However, it is important to remember that we performed relatively shallow sequencing and that studies that require deeper sequencing will likely need a larger quantity of complex libraries. Furthermore, our samples were relatively young, mostly from the 19th and 20th centuries (Table S1), and DNA degradation increases with specimen age (Zimmermann et al. 2008; Sawyer et al. 2012)—albeit not consistently (Sawyer et al. 2012)—as we also observe in our analysis (Table S6). Therefore, the optimal amount of dental calculus depends on the requirements of each specific study.

By better understanding the inhibition mechanism, researchers could develop more robust methods for dental calculus metagenomics, for example, by relying on inhibition‐tolerant DNA polymerases (Sidstedt et al. 2015), amplification facilitators (Wilson 1997), additional inhibitor removal steps, for example, Zymo OneStep Inhibitor Removal Kit (Capo et al. 2021), or alternative library preparation methods, as long as these approaches are compatible with degraded and chemically modified molecules typical of historical and ancient samples.

In addition, we found that dental calculus from different species varied in the amount of DNA that could be extracted from it. Animalivores had a higher DNA yield relative to sample weight, with 0.042 μg of DNA per mg of dental calculus, compared to 0.015 μg for a typical herbivore. This is a considerably lower yield than, for example, modern dental pulp (~0.200 μg/mg), but for animalivorous host species, it is comparable to that of blood (~0.030 μg/mg) (Siuta et al. 2024). It is not clear why host species differ in their DNA yield, but it may relate to the structure and chemical composition of dental calculus. As a living biofilm, dental plaque (the uncalcified form of dental calculus) consists of bacterial cells that are embedded within a secreted extracellular matrix (Schroeder and Shanley 1969). The composition of this biofilm, and specifically the matrix‐to‐cell ratio, can vary, even within humans (Frank and Brendel 1966). It is reasonable to expect similar or greater variation across host species included in this study, and a distinct dental calculus composition, possibly more “densely packed”, with a greater cell to matrix ratio, may explain the increased DNA yield in animalivorous host species.

Although we do not fully understand which factors lead to changes in dental calculus structure, dietary habits, oral microbiota, and salivary composition are plausible candidates. Salivary composition varies across mammals, including the content of glycoproteins (Herp et al. 1979), which are key nutrients for early plaque bacteria (Jakubovics 2015). Dietary carbohydrates are also important, but in later stages of plaque development (Jakubovics 2015; Jakubovics et al. 2021; Frank and Brendel 1966). Biofilm formation is performed by the oral microbiota, which starts by adhering to each other and the tooth surface and then secretes the extracellular matrix. Although speculative at this stage, salivary composition, specific members of the oral microbiota, or the carbohydrate‐poor diet of animalivorous mammals (Figure S1) may contribute to a denser dental calculus structure. Further experiments, e.g., using electron microscopy to investigate dental calculus structure in different mammalian hosts or evaluating the ability of microbiota to secrete the extracellular matrix, are needed to provide more definitive explanations. Alternatively, the increased DNA yield in animalivores could reflect inhibition during DNA fluorometry‐based quantification, which may lead to underestimation of DNA concentration in frugivores and herbivores (Zipper et al. 2003; Sidstedt et al. 2015). Dental calculus tends to be rarer in wild animalivores than in herbivores and, if present, is found in lower quantities (Richards et al. 2025); this means a higher DNA yield in animalivores is encouraging, as it suggests that less material can be used to obtain sufficient DNA for downstream applications.

Dental calculus is known to entrap host and dietary molecules (Mann et al. 2018, 2020; Warinner et al. 2015). However, the initial excitement about the possibilities of studying host genomics and diet from this material has been subdued after the realisation that eukaryotic reads account for < 1% of DNA obtained from human dental calculus (Mann et al. 2018). Although higher host content was detected in non‐human mammals (from 0 up to 74%; Brealey et al. 2020; Moraitou et al. 2022), so far most host genomic analyses from dental calculus samples have primarily focused on mitochondrial DNA (but see Brealey et al. 2020). Our results suggest variation in host content across mammalian orders, with a lower proportion in primates and a higher proportion in elephants and rodents (Table S8, Figures S8 and S9).

However, these results should be interpreted with caution, as there is a large variation in morphology of dental calculus across host species and individuals (Richards et al. 2025), which impacts the ease of sampling. Chunky calculus deposits, such as those frequently seen in omnivores and animalivores (and some herbivores, Figure 1), can be removed by applying pressure at the base of the deposit, whereas filmy deposits require scraping of the tooth surface, thus increasing the probability of co‐sampling host tooth tissues. The sampled elephants tended to have thinner dental calculus deposits (Richards et al. 2025) than other taxa. Therefore, during sampling, more tooth tissue may have inadvertently been collected alongside the calculus. Similarly, because of the small size of rodents, more teeth had to be sampled, again increasing the exposure of underlying tooth tissue and potentially leading to higher host read proportions. On the other hand, the lower host content in primates may be the result of a technical artefact during bioinformatic analysis. Specifically, to remove host reads from the dental calculus metagenomes, we performed competitive mapping, using a concatenated reference of the host and the human genome (the latter used as the most likely contaminant during museum storage, handling, and laboratory processing). However, the genetic similarity between human and primate host may lead to increased removal of primate reads that map to the human genome, particularly if the quality of the primate reference is low. Such instances will be less frequent for host species that are evolutionarily more distant from humans, for example, carnivorans.

A similar explanation may also apply as to why primates show by far the highest oral proportion (Figure 6, Figure S10). Although we included oral metagenomes from non‐human mammals as sources for decOM to counteract the human bias of the default sources, most of the oral sources were still from human samples, simply because wild animal oral metagenomes, and particularly dental plaque or calculus, are very scarce. This may explain why, overall, the ‘human oral’ fraction appears on average larger than the ‘terrestrial mammal oral’ fraction (Figure 6, Figure S10). Primates have the highest total oral fraction, which is almost entirely comprised of human oral metagenomes (Figure S10). This observation agrees with previous studies that show that the primate oral microbiome is fairly conserved (Asangba et al. 2022; Fellows Yates et al. 2021). On the other hand, the two species with the highest ‘unknown’ proportion in their microbiomes are both marine: the South American sea lion ( *O. flavescens* ) and the dugong ( *D. dugon* ). Although we included oral metagenomes from marine mammals, those represented a different source, dental sulcus, and all but three were amplicon data (Dudek et al. 2017), which may explain why they account for a small proportion of dental calculus metagenomes, even those of marine mammals in our dataset (although the orca and the sea lion had the highest proportion explained, see Figure 6; Figure S10). This is yet another reminder of the considerable gaps in omics databases that are so often used as reference, and highlights the need to expand the characterisation of the animal microbiomes to a wider phylogenetic and ecological diversity and to develop and embrace methods that do not rely solely on reference databases (Worsley et al. 2024; Leonard et al. 2024), such as de‐novo assemblies.

Our results highlight several key considerations relating to dental calculus samples (summarised in Table 3) that we hope will be useful for researchers and curators alike when planning future projects. First, despite the considerable diversity in diet, lifestyle, tooth morphology and many other ecological and evolutionary traits, the mammals included in this study all produced dental calculus deposits with a clear oral microbiome content. This suggests that the study of oral microbiome evolution and many other questions in ecology and evolution can be performed using dental calculus across a diverse set of mammalian host species. Second, dental calculus from hosts with herbivorous diets tends to be enriched in enzymatic inhibitors (Figure 4, Figures S2 and S4). Therefore, special considerations must be put in place when initiating studies of the herbivore dental calculus microbiome. These could include optimisations of extraction protocols that can more efficiently remove inhibitors, the use of robust DNA polymerases during library preparation, and pilot studies that allow estimating success rates for specific focal species to inform sampling strategies and sample sizes. We find that, for samples with high inhibitor load, using more than 10 mg of dental calculus is unlikely to improve the success rate of the laboratory procedures or increase the proportion of unique reads in the metagenomic libraries. Therefore, there is little benefit to sampling more than 10 mg (an amount a bit larger than a linseed, according to a guide by Warinner and Fagernäs (n.d.)), which could reduce sampling pressure on the museum specimens, preserving more of the dental calculus material for future studies (Austin et al. 2024). Third, although the incidence of dental calculus in animalivorous species is low (Richards et al. 2025), the deposit appears to contain more DNA (Figure 3) and therefore a smaller sample amount is likely to be sufficient. These findings can guide dental calculus sampling and library preparation, facilitating the study of wild microbiomes from the past and present, whereas at the same time, minimising the pressure of destructive sampling on museum collections.

**TABLE 3:** Summary of factors impacting the preparation of metagenomic libraries, indicating positive, negative or no effects, alongside key considerations and recommendations for researchers.

	DNA extraction	Library preparation (barcoding and indexing)
Host diet	Animalivory ↑ (positive)	Herbivory ↓ (negative)
Extract pigmentation	0 (no effect)	↓ (negative)
Specimen age	↓ (negative)	0 (no effect)
Key considerations and recommendations	Animalivores show a higher DNA yield: less dental calculus material may be required. Sampling amount could be adjusted to offset DNA degradation with age.	Dilution (when DNA concentration allows it) may reduce inhibition. Inhibitor removal steps or alternative enzymes may be considered. Consider compatibility with ancient DNA. Robust methods for dental calculus metagenomics are needed.

## Author Contributions

K.G., M.M. and J.R. conceived the study. M.M. and J.R. collected samples and performed laboratory work. M.M. analysed the data and produced figures, with contributions from C.B. F.E.Z., K.S., E.G., Z.T., A.C.K., O.P., R.S., P.K. and R.P.M. provided logistical support and access to samples and curated sample metadata. M.M. wrote the manuscript with input from K.G. and all co‐authors. K.G. administered the project, acquired funding and resources.

## Disclosure

Benefit sharing statement: All samples included in this study were obtained from historical specimens housed in European natural history collections. Sample collection and processing methodologies were approved by the respective curators prior to the commencement of the study.

## Conflicts of Interest

The authors declare no conflicts of interest.

## Supporting information


**Figure S1:** men70039‐sup‐0001‐Figures.pdf.

**Table S1:** men70039‐sup‐0002‐Tables.xlsx.

## Data Availability

All scripts used for the pre‐processing of metagenomic sequences and for the statistical analysis (models, machine learning and plots), as well as specimen and laboratory‐related metadata are available at: https://github.com/markella‐moraitou/mammal_dentalcalculus_methods/. Sequencing data have been deposited in the European Nucleotide Archive under project accession number PRJEB91453.
