# Supplementary material for: Host Traits Impact the Outcome of Metagenomic Library Preparation From Dental Calculus Samples Across Diverse Mammals
Source: Mol Ecol Resour. 2025 Sep 1;25(8):e70039. doi: 10.1111/1755-0998.70039 (PMC12550474; doi:10.1111/1755-0998.70039)
Supplement: Supplementary file 1 — Figure S1: men70039‐sup‐0001‐Figures.pdf. [file MEN-25-e70039-s002.pdf]

MOLECULAR ECOLOGY RESOURCES

Supplemental Information for:

Host Traits Impact the Outcome of Metagenomic Library Preparation from Dental Calculus Samples Across Diverse Mammals

Markella Moraitou[1], John L. Richards[1], Chanah Bolyos[1], Konstantina Saliari[2], Emmanuel Gilissen[3,4], Zena Timmons[5], Andrew C. Kitchener[5,6], Olivier S. G. Pauwels[7], Richard Sabin[8], Phaedra Kokkini[8], Roberto Portela Miguez[8], Katerina Guschanski[1,9]

1. Institute of Ecology and Evolution, School of Biological Sciences, University of Edinburgh, Edinburgh, United Kingdom
2. Natural History Museum Vienna, Vienna, Austria
3. Royal Museum for Central Africa, Tervuren, Belgium
4. Université Libre de Bruxelles, Alzheimer and other tauopathies research group, ULB Center for Diabetes Research (UCDR), Brussels, Belgium
5. Department of Natural Sciences, National Museums Scotland, Edinburgh, United Kingdom
6. School of Geosciences, University of Edinburgh, Edinburgh, United Kingdom
7. Royal Belgian Institute of Natural Sciences, Brussels, Belgium
8. Natural History Museum London, London, United Kingdom
9. Department of Ecology and Genetics/Animal Ecology, Uppsala University, Uppsala, Sweden

Table of Contents:

**Supplementary Figure 1**	Page 2
**Supplementary Figure 2**	Page 3
**Supplementary Figure 3**	Page 4
**Supplementary Figure 4**	Page 5
**Supplementary Figure 5**	Page 6
**Supplementary Figure 6**	Page 7
**Supplementary Figure 7**	Page 8
**Supplementary Figure 8**	Page 9
**Supplementary Figure 9**	Page 10
**Supplementary Figure 10**	Page 11

MOLECULAR ECOLOGY RESOURCES

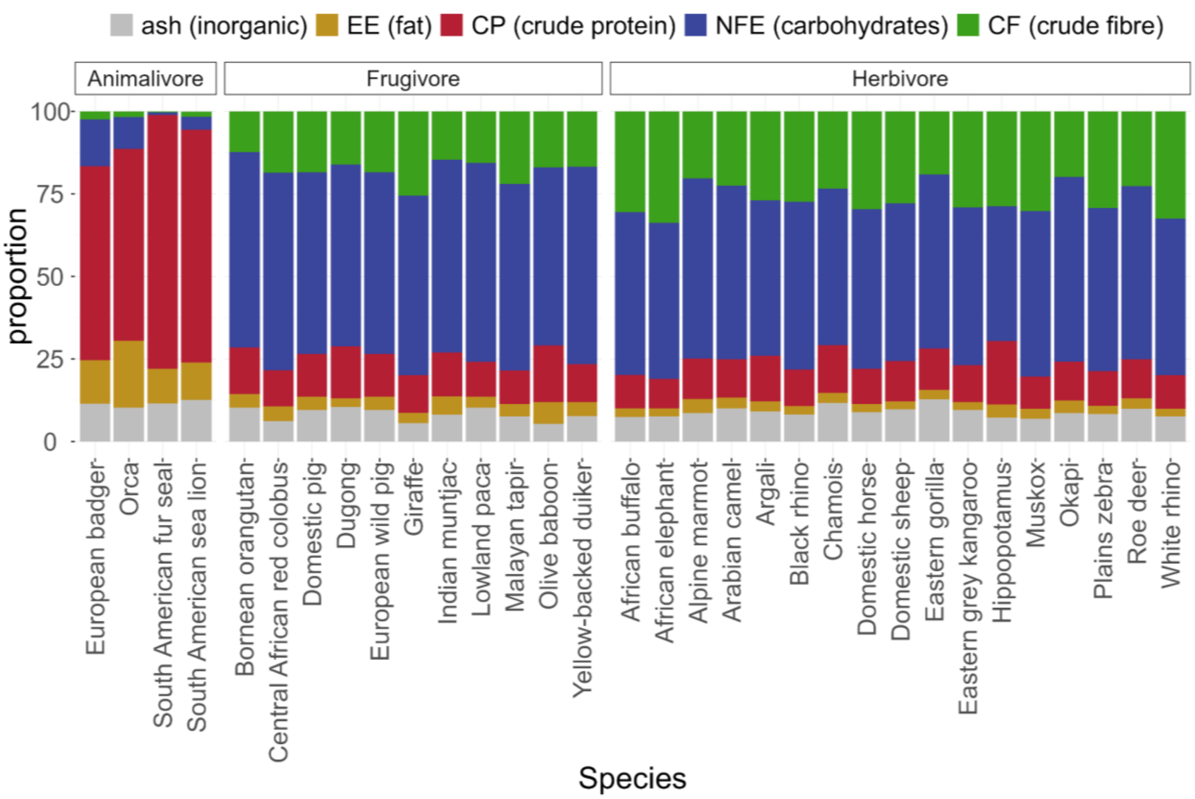

Supplementary Figure 1. Barplot showing chemical composition of the diet of study species according to Lintulaakso et al. (2023) (data in Supplementary Table 2).

MOLECULAR ECOLOGY
RESOURCES

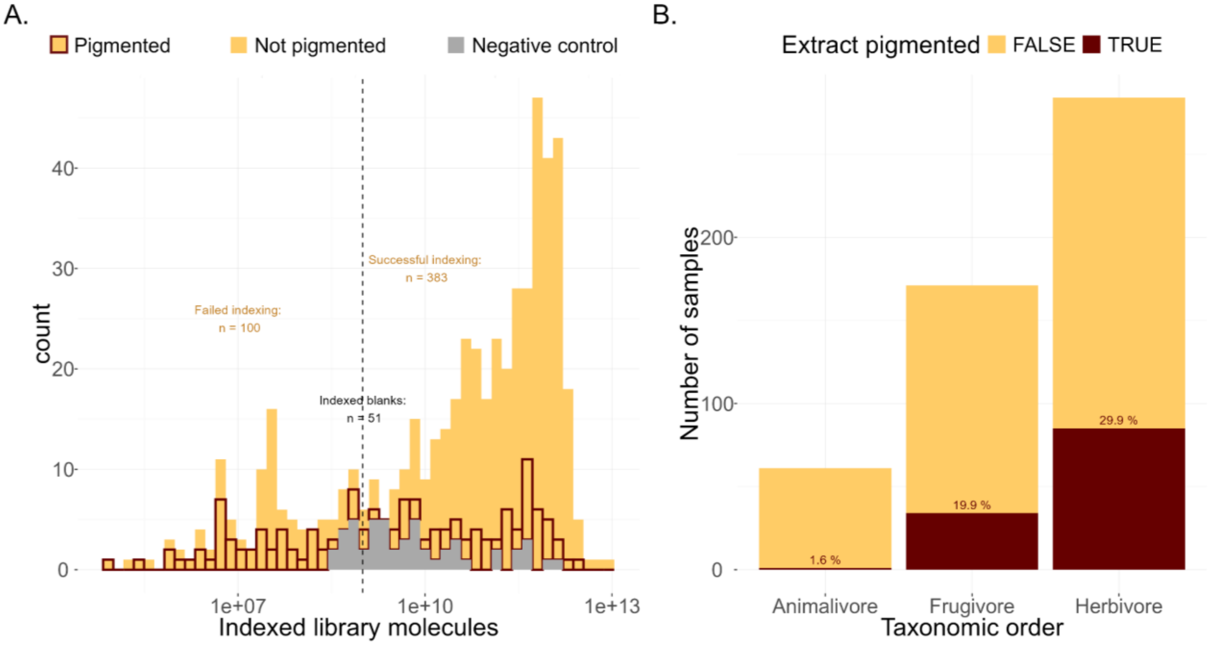

Supplementary Figure 2. A) Distribution of indexed library molecules for samples (yellow) and blanks (grey) (note the log10 scale on the x-axis). Brown outlines show libraries from pigmented extracts. The vertical dashed line at 10^9 indexing copies was used as a cut-off for a successful indexed library and corresponds to ca. the first quartile of indexed library molecules for the negative controls ($1.2x10^9$). B) Proportion of samples in our dataset that generated a pigmented DNA extract, per dietary category, indicating possible co-elution of inhibitors.

MOLECULAR ECOLOGY
RESOURCES

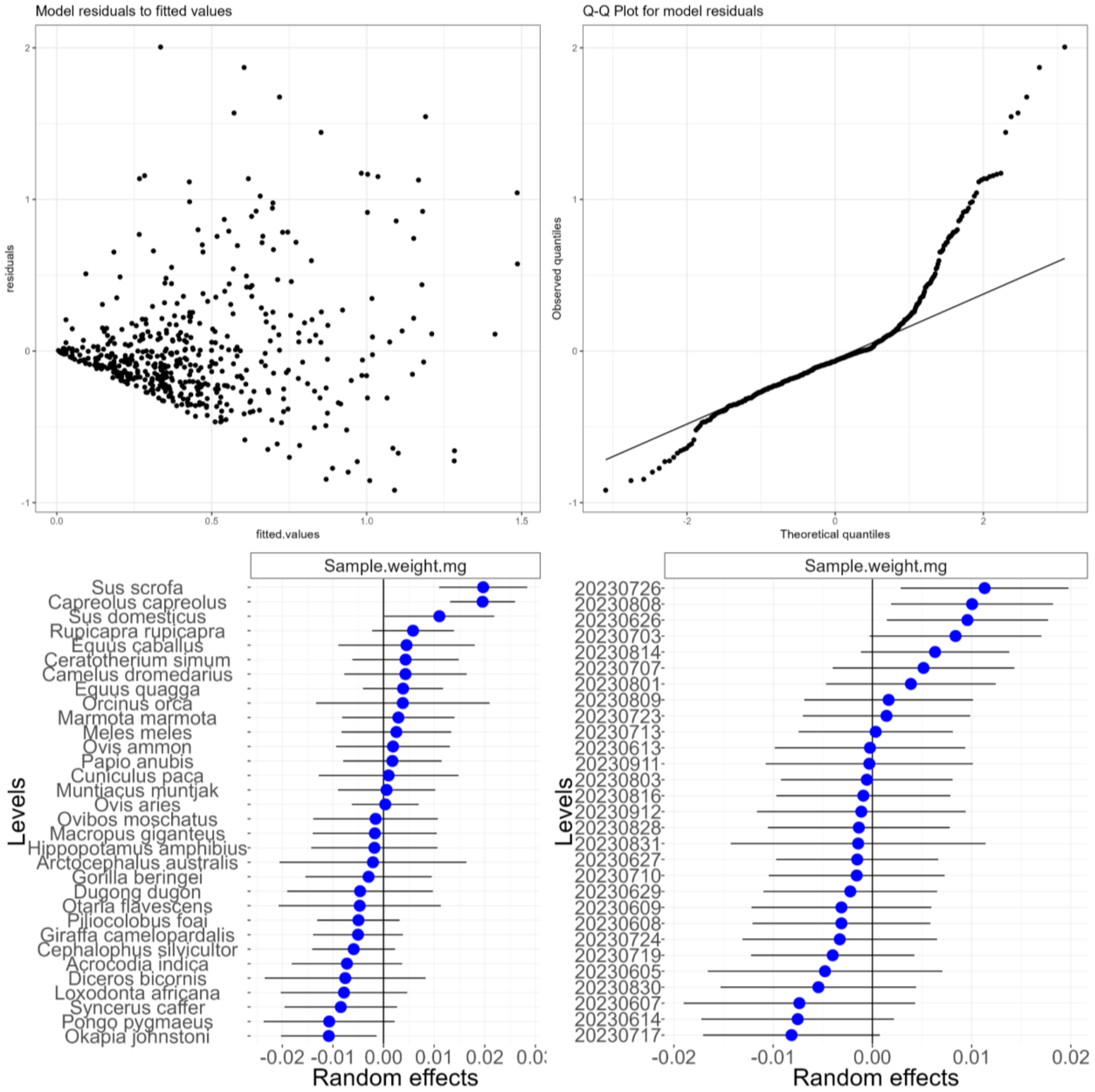

Supplementary Figure 3. Diagnostic plots and random effects for Model 1. Top left: Plot of residuals to fitted values. Top right: Quantile-Quantile (QQ) plot for model residuals. Bottom: left: Random effects for mammalian host species. Bottom right: Random effects for DNA extraction batch.

MOLECULAR ECOLOGY RESOURCES

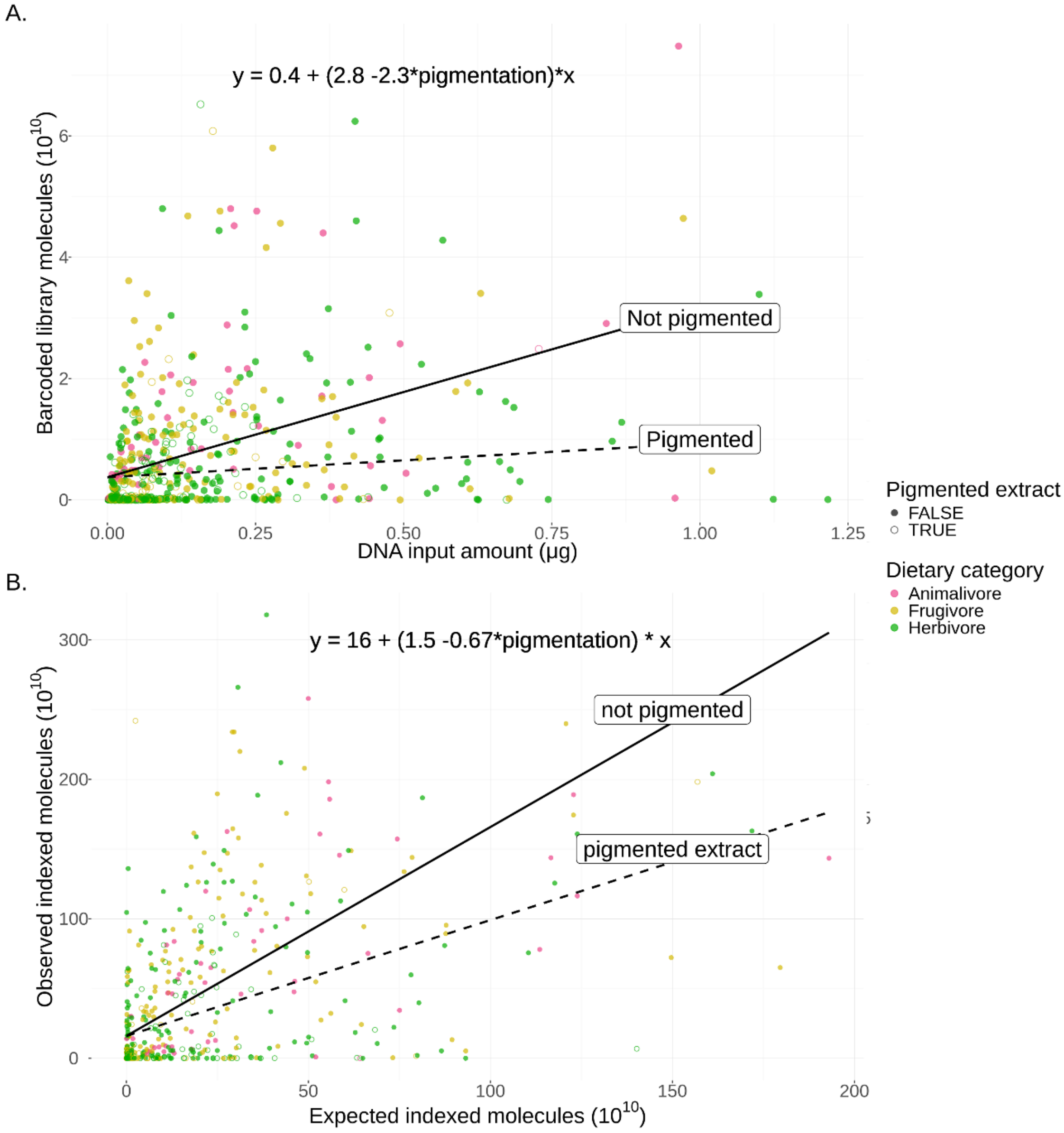

Supplementary Figure 4. A) The relationship between DNA input in µg and barcoded library output for 497 samples that underwent barcoding, and B) the relationship between expected and observed indexed library output for 472 samples that underwent indexing. The formulas on the top are based on all significant model estimators (in both cases, those are the intercept, the input and extract pigmentation) and the two lines show the fitted values for samples with pigmented (dashed) or nonpigmented (solid) DNA extracts according to a linear mixed model.

MOLECULAR ECOLOGY
RESOURCES

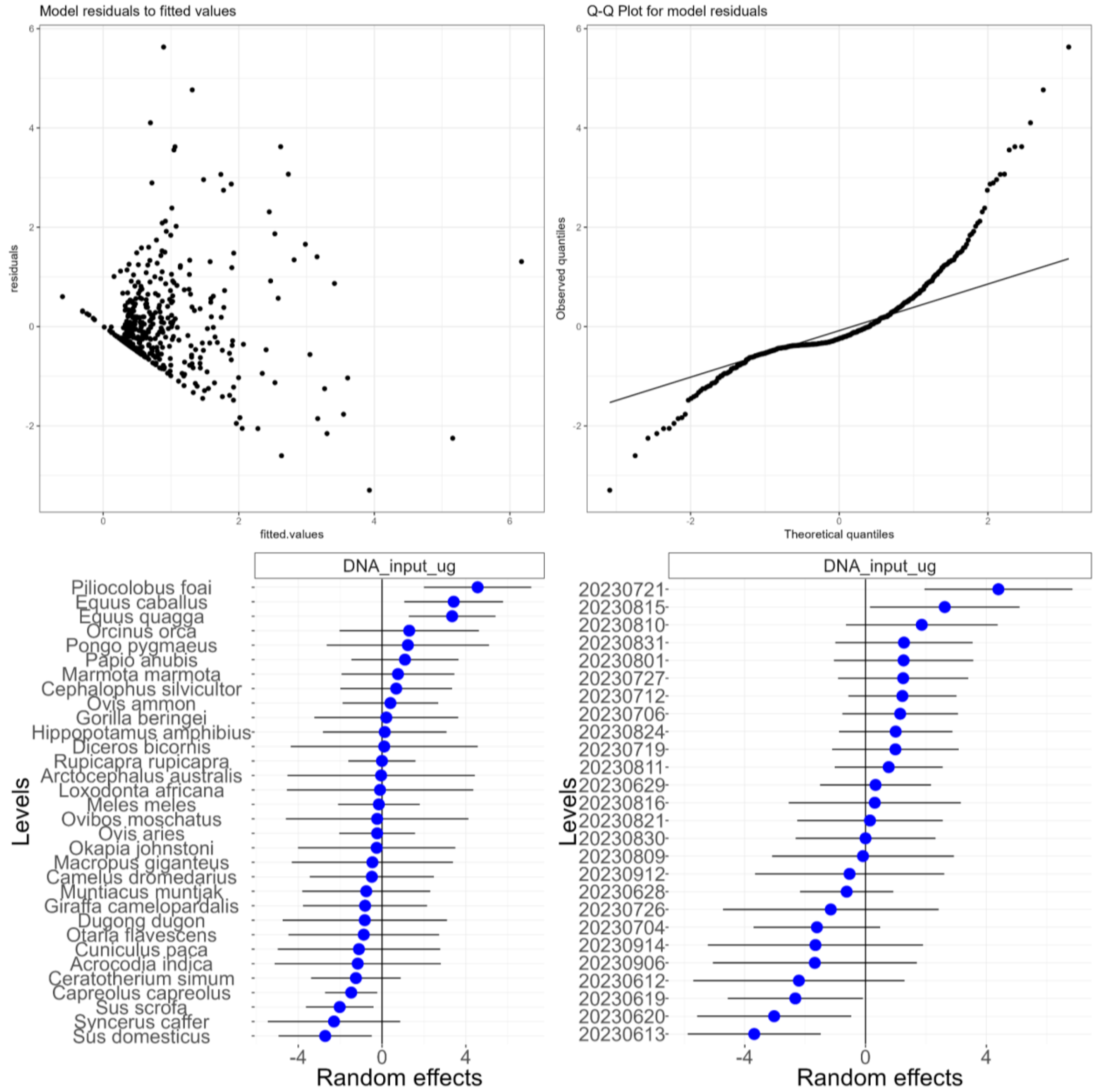

Supplementary Figure 5. Diagnostic plots and random effects for Model 2. Top left: Plot of residuals to fitted values. Top right: Quantile-Quantile (QQ) plot for model residuals. Bottom: left: Random effects for mammalian host species. Bottom right: Random effects for adapter ligation batch.

MOLECULAR ECOLOGY
RESOURCES

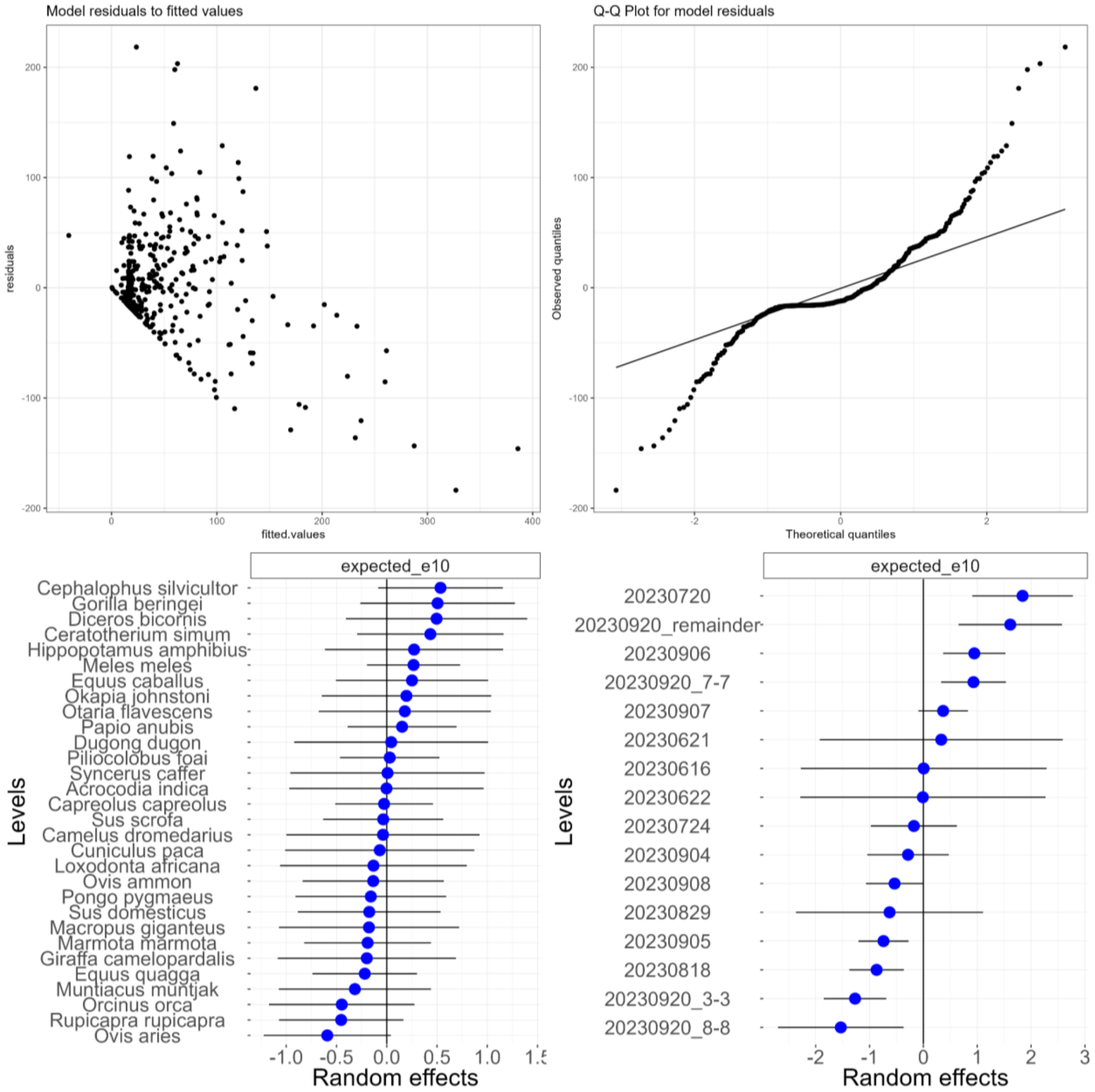

Supplementary Figure 6. Diagnostic plots and random effects for Model 3. Top left: Plot of residuals to fitted values. Top right: Quantile-Quantile (QQ) plot for model residuals. Bottom: left: Random effects for mammalian host species. Bottom right: Random effects for indexing batch.

MOLECULAR ECOLOGY RESOURCES

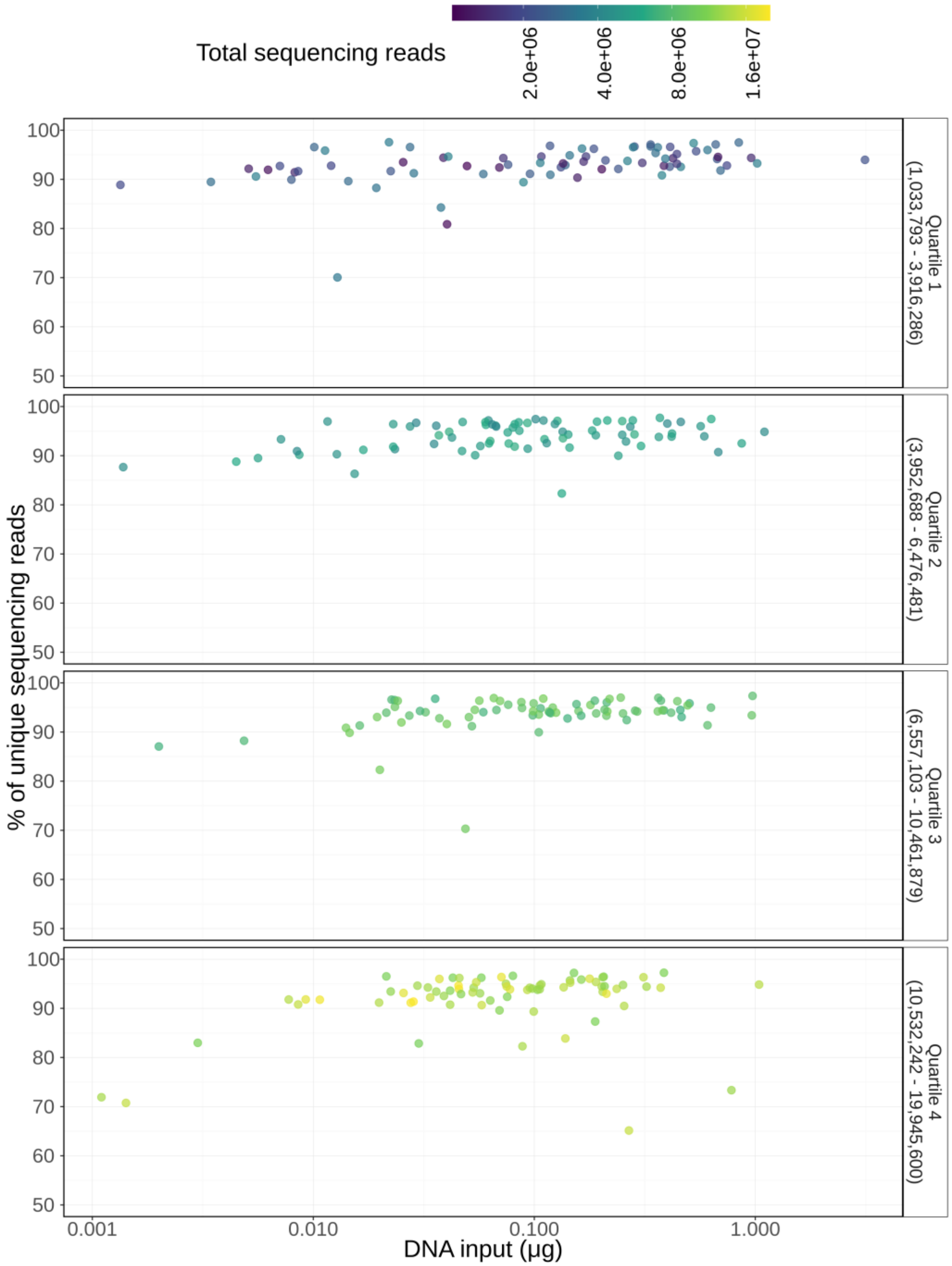

Supplementary Figure 7. Percentage of unique sequencing reads plotted against the initial DNA input (in µg) used for library preparation presented separately for each quartile of sequencing depth. Like in Figure 5 in the main text, this visualisation includes 302 samples with sequencing depth between 10^6 and 2×10^7 reads. Note the log10-transformed x-axis and colour scale.

MOLECULAR ECOLOGY RESOURCES

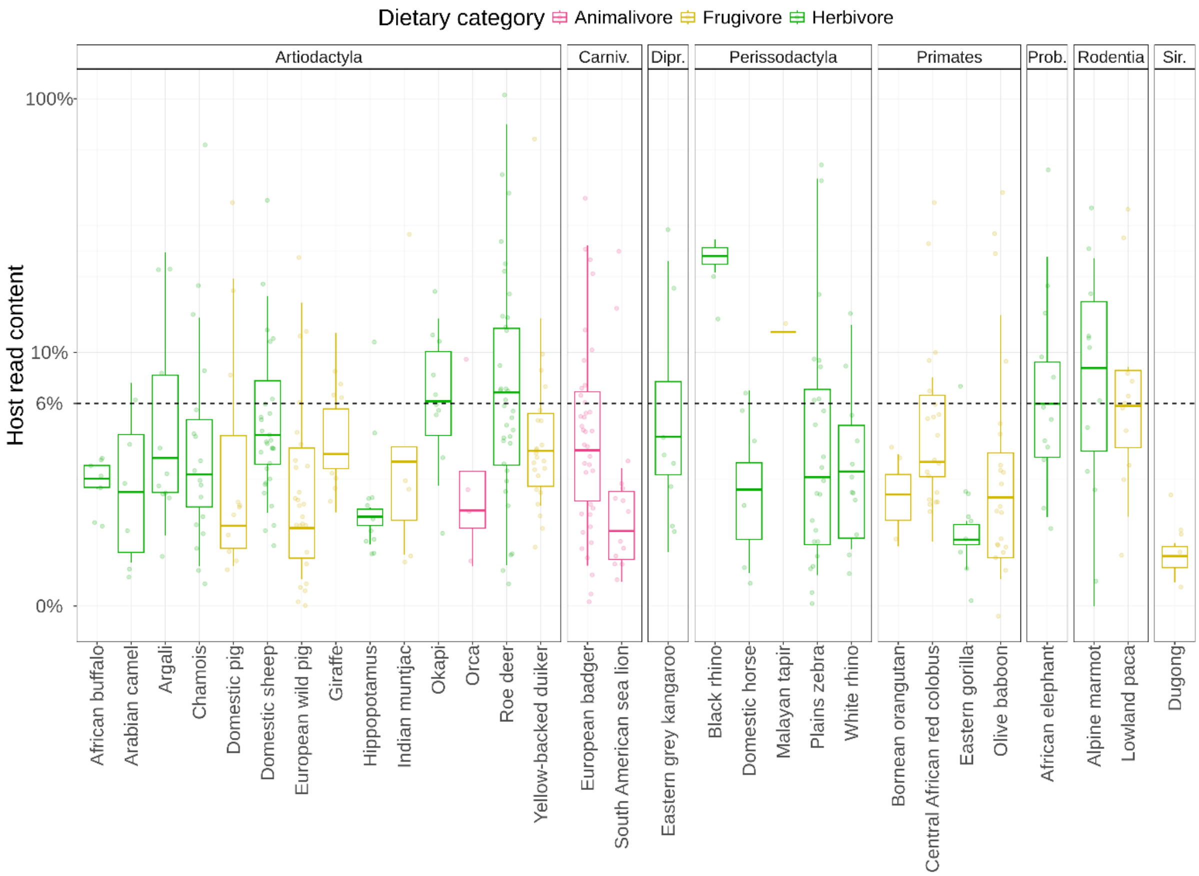

Supplementary Figure 8. Percentage of host reads in dental calculus for different species. Colour indicates dietary category based on Lintulaakso et al. (2023). Facets reflect different taxonomic orders. Note the log10-transformed y axis to increase the visibility of low values. Abbreviations: Carniv. = Carnivora, Dipr. = Diprotodontia, Prob. = Proboscidea, Sir. = Sirenia. The horizontal dashed line indicates the average host read percentage (5.9%).

MOLECULAR ECOLOGY RESOURCES

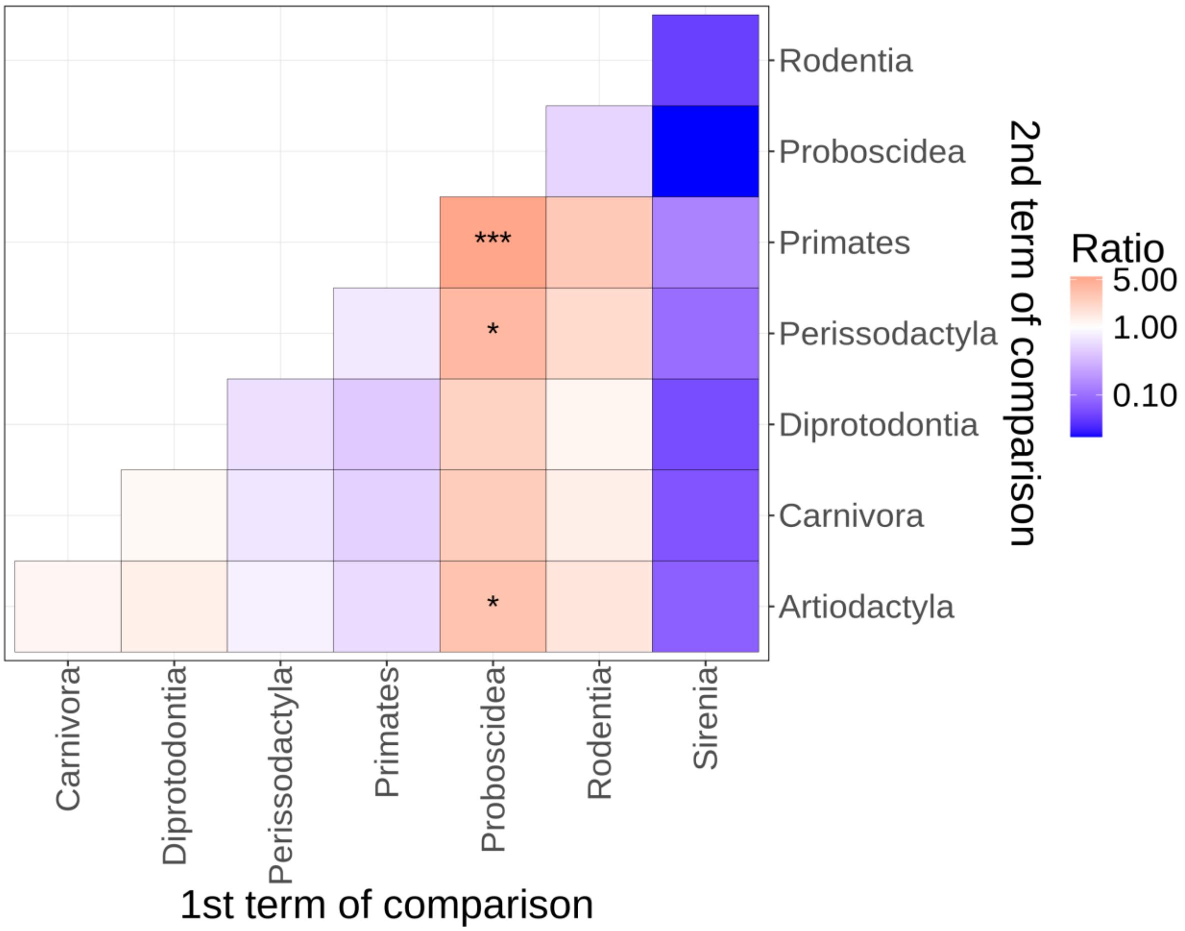

Supplementary Figure 9. Ratio of host DNA content (%) between orders. Red indicates that the host taxonomic order on the x-axis has a larger proportion of host reads than the order on the y-axis, while blue indicates the reverse. Significance levels: (***) $p < 0.001$, (**) $p < 0.01$, (*) $p < 0.05$, (.) $p < 0.1$.

MOLECULAR ECOLOGY RESOURCES

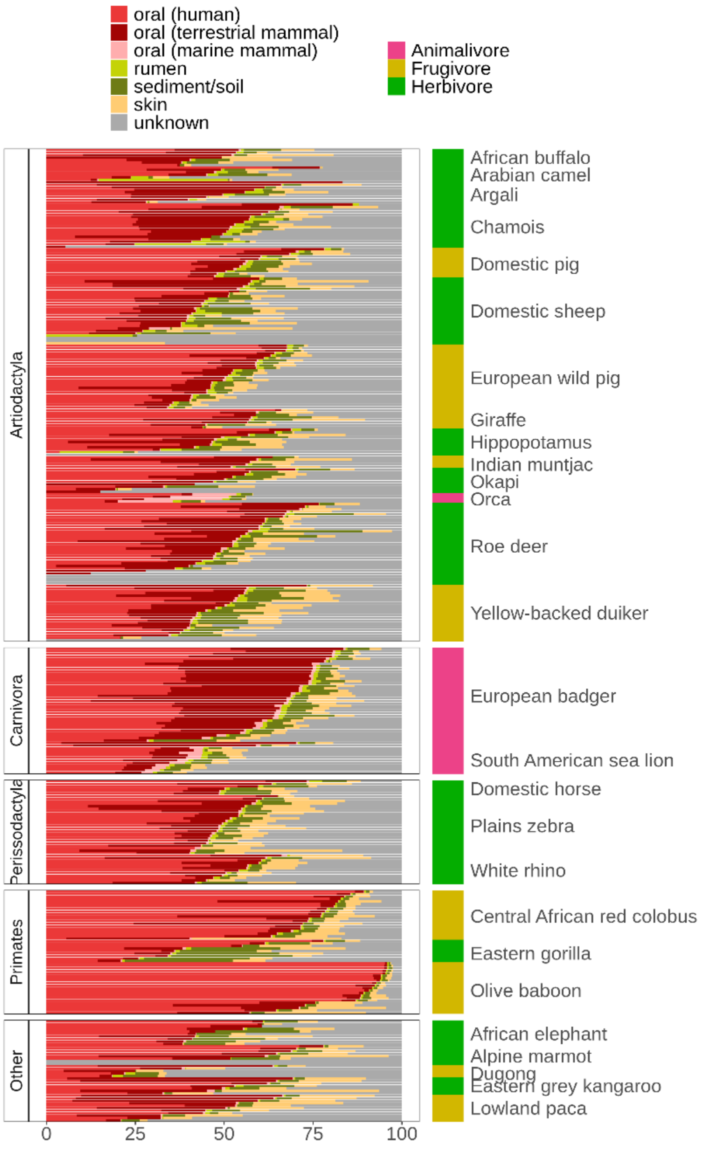

Supplementary Figure 10. Estimated composition of dental calculus metagenomes (after removing reads mapping to the host and human genome) with regard to four types of microbiome sources: human ancient oral, human modern oral, human skin and sediment/soil. The proportions were estimated using a k-mer based approach implemented in decOM (Duitama González et al. 2023). The “Unknown” partition refers to sequences that were found in the samples but none of the sources, however it's a feature under development and may not be entirely accurate. The bar on the right side indicates the dietary category of the mammalian host. Plot data can be found in Supplementary Table 3.
